# Mental Health Supports for SOGIE Asylum Seekers in Canada: A Community-Responsive Scoping Review

**DOI:** 10.3390/bs16091484

**Published:** 2026-08-25

**Authors:** Aaron Yan-Pui So, Taymy Josefa Caso, Sophie Yohani

**Affiliations:** Faculty of Education, University of Alberta, 11210-87 Avenue NW, Edmonton, AB T6G 2G5, Canada; caso@ualberta.ca (T.J.C.); sophie.yohani@ualberta.ca (S.Y.)

**Keywords:** SOGIE, LGBTQ, asylum seeker, refugee claimant, mental health, Canada, scoping review

## Abstract

While research has investigated the mental health burdens of refugee and asylum seekers with diverse sexual orientation and gender identity and expression (SOGIE), far less attention has been given to what supports their mental health, particularly within the Canadian context. In response to an identified need from a local community organization, this scoping review aimed to identify current approaches that support SOGIE asylum seekers’ mental health in Canada. Using the PRISMA-ScR guidelines, searches took place on 7 and 8 March 2026 across peer-reviewed and grey literature databases indexed in MEDLINE, EMBASE, PsycINFO, CINAHL Plus, LGBTQ+ Source, Scopus, Web of Science, SocIndex, Sociological Abstracts, ERIC, Global Health, ProQuest Dissertations and Theses Citation Index, EBSCO Open Dissertations, ProQuest’s Canadian Research Index, Government of Canada Library, Canadian Council for Refugees Member Organizations, Newcomer Research Library, UNHCR Canada, Egale, and Google. Title, abstract, and full-text screening were completed by two team members, and seven studies met the eligibility criteria outlined. The analysis of four peer-reviewed studies and three grey literature documents suggests that SOGIE asylum seeker mental health should involve emotional and psychological support during the refugee determination process, as well as address social determinants of health. Additionally, the available literature indicated that there must be an awareness of how stigma and mistrust, intersectional challenges, and structural barriers impact how mental health supports are provided to and received by SOGIE asylum seekers in Canada. While having seven included documents primarily based in Ontario and Quebec is a limitation in the evidence, this also provides evidence for the need to conduct further research about SOGIE asylum seeker mental health in other areas of Canada.

## 1. Introduction

According to Article 3 of the Universal Declaration of Human Rights, “[e]veryone has the right to life, liberty and the security of person” (United Nations General Assembly, 1948). And yet, the United Nations High Commissioner for Refugees (UNHCR) estimates that at the end of 2025, 117.8 million people were forcibly displaced due to persecution and conflict, and nine million were considered asylum-seekers (UNHCR, 2026). Individuals of diverse sexual orientation and gender identity and expression (SOGIE) often flee their country of origin and seek refuge in another country to escape acts of identity-based violence (D’souza et al., 2022). In mid-2026, there were 66 countries that criminalized consensual same-sex acts that occur in private settings, 42 countries that criminalized consensual sexual activity between women, and 13 countries that criminalized trans identity and expression (Human Dignity Trust, 2026). In 12 countries, SOGIE individuals have the possibility of experiencing the death penalty by public stoning, whipping, or other means (Human Dignity Trust, 2026; International Lesbian, Gay, Bisexual, Trans, and Intersex Association [ILGA], 2026).

When an individual flees their country of origin to seek refuge in another country but has not received refugee status, they are known under the international term of “asylum seeker” (Rainbow Refugee, n.d.). Once a SOGIE asylum seeker arrives at a Canadian port of entry and is deemed eligible to make a refugee claim, a claim will be sent to the Immigration and Refugee Board of Canada (IRB; (IRCC, 2021)). The asylum seeker is now considered a refugee claimant and will be released on certain terms and conditions while waiting for a claims hearing (IRCC, 2021). Within the Canadian context, the term “refugee claimant” is used instead of “asylum seeker”, and more specifically signifies an asylum seeker who submitted a refugee claim in Canada (Rainbow Refugee, n.d.). For the purposes of this article, the terms “asylum seeker” and “refugee claimant” will be used interchangeably. Once refugee claimants make their case at a hearing and receive a positive decision, they are now legally protected persons and can work towards becoming a permanent resident of Canada (IRCC, 2021).

This period of preparing for their refugee determination hearing creates high levels of worry and fear for SOGIE refugee claimants (Lee & Brotman, 2011). This process can be retraumatizing and violent for SOGIE individuals because they need to share traumatic details of their life history when they may not be prepared to do so (Alessi et al., 2021). SOGIE refugee claimants only have 45 calendar days after the claim was referred to the IRB to complete and submit a 10-page Basis of Claim document that explains the reason for claiming refugee protection (IRB, 2012, 2025). For some, they may not have documentation from their country of origin to support their asylum claim (Kahn & Alessi, 2018), such as trans refugee claimants unable to change information on their documentation from their country of origin due to safety and security reasons (Lee & Brotman, 2011). As a result, SOGIE refugee claimants must find other ways to prove their sexuality or gender identity to support their asylum claim and to fit identity stereotypes. This can create additional distress and further exacerbate mental health and identity crises (Kahn & Alessi, 2018; Lee & Brotman, 2011).

In addition to the barriers faced in the immigration and refugee determination processes (Lee, 2019), SOGIE refugee claimants experience challenges in meeting their basic needs (e.g., housing, food, employment, and income; Kahn & Alessi, 2018; Mulé, 2022). SOGIE refugee claimants may also be actively managing and experiencing negative impacts on their health and well-being that occurred across the migration journey. They may experience current and past suicidal ideation (Hopkinson et al., 2017), hopelessness (Hermaszewska et al., 2022), post-traumatic stress disorder (PTSD; Attia et al., 2022), anxiety, depression, and substance abuse (Shidlo & Ahola, 2013). Interpersonally, experiences of violence (Alessi et al., 2021), social isolation and loneliness (Yarwood et al., 2022), and discrimination across gender, sexuality, race, and ethnicity often persist while in transit and in the host country (D’souza et al., 2022; Gottvall et al., 2023). However, when SOGIE refugee claimants seek supports for their mental health, the mental health supports they access may not be equipped to address the intersecting experiences of gender identity, sexuality, race, and ethnicity (Murugan et al., 2024).

### 1.1. Current Public Opinion and Rhetoric of Immigration in Canada

Alongside the processes that SOGIE refugee claimants are required to go through as part of the determination process, national sentiment towards immigration within Canada has become increasingly negative when compared to the past few decades (Besco & Goel, 2025). Close to half of Canadians surveyed endorsed the sentiment that too many asylum seekers were being accepted into the country (IRCC, 2025). Along with these negative sentiments being observed, the Government of Canada made changes to the eligibility criteria for asylum claims by passing Bill C-12—Strengthening Canada’s Immigration System and Borders Act into law on 26 March 2026 (IRCC, 2026a). Under Bill C-12, “asylum claims made more than one year after someone’s first entry into Canada” or “who enter Canada between ports of entry along the Canada–US land border and who make a claim after 14 days” will not have their claims referred to the IRB (IRCC, 2026a). In addition, the government has increased power to “cancel, suspend or change a large group of immigration documents, pause application intake, or cancel or suspend application processing” if it is deemed to be in the public interest and now “has clear legal authority to share certain personal information within the department and with domestic government partners” (IRCC, 2026a).

Organizations that focus on civil liberties, law, and refugee services have viewed the Strengthening Canada’s Immigration System and Borders Act as concerning (Canadian Council for Refugees, 2025; Shory, 2025). For vulnerable claimants, such as SOGIE refugee claimants, this Act increases risks for safety (e.g., concerns about one’s gender identity or sexual orientation being shared with others, creating risks for discrimination and abuse) and precariousness (e.g., concerns of sudden termination of documentation; Canadian Council for Refugees, 2025). With regard to the one-year timeframe, organizations have critiqued that this does not account for the unique needs of vulnerable SOGIE refugee claimants (e.g., SOGIE refugee claimants may not feel safe to disclose their identity within the new one-year timeline; Canadian Council for Refugees, 2025; Shory, 2025).

### 1.2. Study Rationale, Objective, and Research Question

Existing research has thoroughly investigated the mental health challenges of SOGIE refugees and asylum seekers worldwide through reviews and syntheses (Alessi et al., 2021; Chaudhry et al., 2024; D’souza et al., 2022; Gottvall et al., 2023; Hermaszewska et al., 2022; Nematy et al., 2023; Yarwood et al., 2022). Mental health supports and interventions have also been investigated for individuals who are refugees and asylum seekers (Herati & Meyer, 2023; Slobodin & De Jong, 2015; Turrini et al., 2017, 2019), and who are SOGIE (Austin et al., 2018; Tudor-Sfetea & Topciu, 2024; Yang et al., 2024). However, there has been less focus on the mental health support and interventions that SOGIE asylum seekers require at the unique intersection of migration status, sexual orientation, and gender identity. Previously, a rapid review investigated the mental health needs of SOGIE refugees within the Canadian context (Murugan et al., 2024); however, this current scoping review will be focused exclusively on SOGIE asylum seekers and will include grey literature to supplement findings from peer-reviewed articles (Adams et al., 2017).

The objective of the scoping review was to answer the following research question: What is currently known about the approaches taken to support the mental health of SOGIE asylum seekers in the Canadian context? In collaboration with community partners at the Edmonton Newcomer Centre (a newcomer-serving organization in Edmonton, Alberta, Canada), we identified the appropriate scope and topic for the scoping review. As such, this represents the collaboration between the research team and community partners to further the understanding of SOGIE asylum seeker mental health in Canada. In addition, information about the included literature was documented in this scoping review (e.g., participant information, methodologies used, theoretical frameworks, and study findings) to identify and map the current landscape of SOGIE asylum seeker mental health research in Canada.

## 2. Materials and Methods

### 2.1. Positionality and Community Engagement

A.Y.-P.S. has conducted various community-based research projects with community partners as part of his undergraduate honors thesis and his numerous research assistant positions. In addition, his interest in working with SOGIE and refugee and asylum seeker populations comes from A.Y.-P.S. being a racialized SOGIE man whose family were refugees. As such, A.Y.-P.S. wanted his master’s thesis (this scoping review) to be community-based, focused on SOGIE and refugee and asylum seeker populations, and be mobilized into the community setting.

T.J.C. identifies as a racialized, disabled, queer, nonbinary, immigrant academic researcher and counselling psychologist. Their research focuses on health disparities, intersectionality, identity-based marginalization within LGBTQ+ BIPOC communities, gender and sexual fluidity, social determinants of health, and legislative and public policy advocacy. They have extensive experience in leading transdisciplinary research projects with community partnerships focused on improving health care systems engagement, codesigning participatory action research projects to address health care access barriers, reducing 2SLGBTQ+ health disparities, codesigning technological development to expand access to care, and developing smart clothing to improve trunk support for people with lived experience of disability.

S.Y. identifies as a racialized woman and migrant living in Treaty 6 territories in Canada. As a psychologist and professor of counselling psychology, her research centres on community-engaged, anti-oppressive, and culturally grounded approaches to mental health promotion, intervention, and equity with migrant and other structurally marginalized populations. With over 30 years of clinical practice with migrant populations, S.Y. has worked extensively and directly with refugees and asylum seekers, while also supervising clinicians providing services to these populations.

Community partners at the Edmonton Newcomer Centre (ENC) were consulted by A.Y.-P.S. and S.Y. in May 2025. During this meeting, community partners identified their needs and challenges related to SOGIE refugee and asylum seeker mental health. A.Y.-P.S. and S.Y. described the scope of this project as being part of A.Y.-P.S.’s master’s thesis, the scoping review methodology, and the expected timeframes of this research project. After summarizing the meeting notes into eight topic areas, A.Y.-P.S. shared the summary with community partners who indicated that any of the topics would support their work. A.Y.-P.S. then explored with S.Y. and T.J.C. the potential topics and, considering feasibility and timelines, chose to investigate what is currently known about the approaches taken to support the mental health of SOGIE asylum seekers in the Canadian context.

### 2.2. Study Design

This scoping review followed the methodological frameworks outlined by Arksey and O’Malley (2005) and Levac et al. (2010). In addition, the Joanna Briggs Institute (JBI) Manual for Evidence Synthesis was referenced to inform the scoping review process and develop the scoping review protocol (Peters et al., 2020). In alignment with goals of improving transparency and reducing biases, the scoping review protocol was pre-registered through the Open Science Framework (So, 2026). The reporting guidelines outlined in the Preferred Reporting Items for Systematic Reviews and Meta-Analyses extension for Scoping Reviews (PRISMA-ScR; Tricco et al., 2018) were followed, with the PRISMA-ScR Checklist provided in Supplementary Materials File S1. Although scoping review methodology does not require critical quality appraisals (Arksey & O’Malley, 2005), the research team still followed the PRISMA-ScR guidelines and engaged in regular meetings and dialogue regarding biases and analytical processes (e.g., coding authenticity, parity between codes, determining scope of interpretation, and other ethical considerations). Decisions were made through research team consensus.

### 2.3. Search Strategy

A.Y.-P.S. conducted searches between 7 March 2026 and 8 March 2026 for peer-reviewed and grey literature. Peer-reviewed databases included Medline (via OVID), EMBASE (via OVID), PsycINFO (via OVID), CINAHL Plus (via EBSCO), LGBTQ+ Source (via EBSCO), Scopus (via Elsevier), Web of Science (via Clarivate), SocIndex (via EBSCO), Sociological Abstracts (via ProQuest), ERIC (via EBSCO), and Global Health (via OVID). Grey literature databases that were searched focused on dissertations (ProQuest Dissertations and Theses Citation Index, EBSCO Open Dissertations), government reports (ProQuest’s Canadian Research Index, Government of Canada Library), and non-profit and advocacy organization websites (Canadian Council for Refugees Member Organizations, Newcomer Research Library, UNHCR Canada, and Egale), along with Google. The databases searched were determined after collaborative discussions with a university librarian who specializes in review strategies and protocols, along with S.Y. and T.J.C.

The search strategy for peer-reviewed articles across databases used the Population–Concept–Context (PCC) framework and the MEDLINE query string for keywords, abstracts, headings, or titles in Table 1. The keywords and terms for the search strategy were developed in consultation with S.Y., T.J.C., and the university librarian. The terms for the NOT search query string were based on terms identified in the preliminary search that would narrow and refine the search results. The feasibility and validation of the search strategy were developed in collaboration with the university librarian. Query strings for other interfaces were translated using the PolyGlot Search Translator that supports translation across different interfaces for peer-reviewed literature (Clark et al., 2020). Search strategies for other databases are provided in Supplementary Materials File S2.

When searching for grey literature, a combination of the following terms was used: “diverse sexual orientation, gender identity and expression OR SOGIE OR LGBTQ” AND “asylum seeker OR asylum claimant” AND “mental health”. During the grey literature search process, “SOGIE” was an uncommon term that consistently yielded zero results. Therefore, the search term “LGBT asylum” was used for most of the search to narrow relevant results. Search terms were used in Google, and the first five pages or 50 results were reviewed. The rationale for the number of results and pages to review for the Google search was based on having enough searches to locate grey literature while also reducing potential redundancies that already appeared in the peer-reviewed literature search. Supplementary Materials File S3 provides a table (adapted from Stapleton, 2018) outlining the search terms used, websites and browsers searched, and results found. Snowball searches were conducted with articles at the full-text screening stage to determine additional literature. Due to resource and time constraints of the project, repeat searches and author contacting did not occur.

### 2.4. Inclusion and Exclusion Criteria

#### 2.4.1. Peer-Reviewed Literature

Peer-reviewed literature was included in the scoping review if it met the following criteria:

- Population: Focused on SOGIE asylum seekers and refugee claimants;
- Content: Focused on mental health and wellbeing;
- Context: Occurred within the Canadian context after 1991 (inclusive) and onward;
- Sources of information: Peer-reviewed empirical journal articles that had full-text access, were written in English, and used either quantitative, qualitative, or mixed methods were included.

For the population and content criteria, the literature that had at least one distinguishable finding that specifically addresses the mental health of SOGIE asylum seekers was also included. The timeframe of 1991 in the context criterion was selected because Canada had begun accepting asylum claims based on persecution of sexual orientation and gender identity that year (LaViolette, 2009).

Peer-reviewed literature was excluded if it did not address SOGIE asylum seeker mental health or well-being (e.g., only focused on refugee or SOGIE populations), or had indistinguishable findings between SOGIE refugee and asylum seeker experiences. Additional exclusion criteria were that the literature was based outside of Canada, was written in a language other than English, was published before 1991, and the full-text document was unavailable or could not be accessed. Lastly, book chapters and other reviews (e.g., scoping reviews, systematic reviews, meta-synthesis, umbrella reviews) were excluded to ensure the articles were empirical and were reviewed by others in the field.

#### 2.4.2. Grey Literature

The inclusion of grey literature broadens the catchment of information that fills some gaps within academic literature (Adams et al., 2017). In addition to the population, content, and context criteria listed for peer-reviewed literature, the following criteria for sources of information were used as inclusion criteria for grey literature:

- Sources of information: Doctoral dissertations, government documents and reports, non-profit and advocacy organization reports, or information from government websites were included.

Inclusion of doctoral dissertations allowed for greater scope for topic search and rigour than master’s theses. Inclusion of government reports, reports from nonprofit and advocacy organizations, and information from government websites can provide greater depth and reduce publication bias (Adams et al., 2017). These sources are also moderately credible (Adams et al., 2017), and they may address the niche needs of SOGIE asylum seekers.

Exclusion criteria for grey literature were the same as the peer-reviewed literature regarding the population, content, geography, timeframe, language, and accessibility. A specific exclusion criterion for grey literature was the sources of information, in that master’s theses, conference posters and proceedings, abstracts, and posts on blogs and social media were excluded. This was because of the lack of information contained and the potential for lower credibility and rigour for consideration (Adams et al., 2017).

### 2.5. Screening and Selection Process

The initial search across databases was uploaded into Covidence, and duplicates were identified manually and by Covidence. A.Y.-P.S. and Shaima Ahammed Thayyilayil (a research assistant; S.A.T.) were the two screeners involved throughout the screening process, under the supervision of S.Y and T.J.C. They first completed pilot screening, where they independently screened ten citations within Covidence using the inclusion and exclusion criteria listed previously. An 80% inter-rater agreement was achieved, with the two disagreements related to grey literature involving programme descriptions from community organizations. After some discussions, A.Y.-P.S. and S.A.T. determined that programme descriptions were excluded because they did not qualify as a programme report and did not contain sufficient rigour.

**Figure 1:**
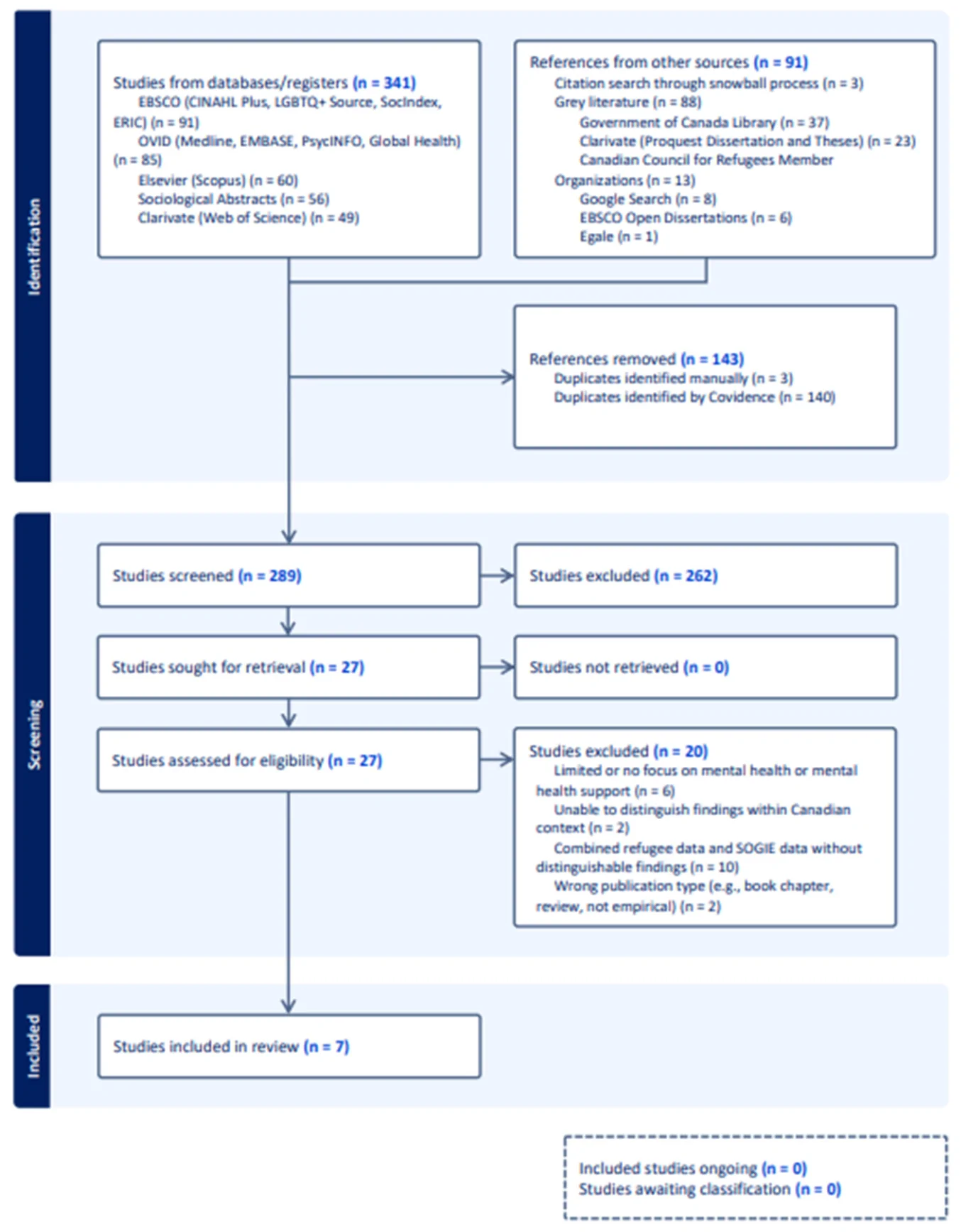
Preferred Reporting Items for Systematic Reviews and Meta-Analyses extension for Scoping Reviews (PRISMA-ScR) flow diagram, as adapted from Tricco et al. (2018).

A.Y.-P.S. and S.A.T. proceeded to independently screen titles and abstracts using the inclusion and exclusion criteria previously outlined. Full-text screening was also independently completed by the same two screeners using the provided inclusion and exclusion criteria. References from these full-text articles were also reviewed to determine if other literature needed to be included. Disagreements during the title/abstract and full-text screening decisions did occur; however, these disagreements were fully resolved through discussions between the two screeners, and a third reviewer did not need to be consulted. The process of study selection is shown in the PRISMA-ScR flow diagram (Figure 1).

### 2.6. Charting and Extraction

A.Y.-P.S. completed extraction for literature that met criteria. S.Y. and T.J.C. reviewed the extraction work to ensure accuracy and completeness. A Google Sheets spreadsheet was created for the charting process. Based on guidance from the JBI Manual for Evidence Synthesis (Peters et al., 2020), the charting table was piloted, and revisions were made via consultation between the three authors.

The finalized charting table captured details related to literature information (title, year of publication, aim and objectives), participant demographics (number of participants, age, country of origin, gender identity, sexual orientation, location in Canada), publication research design (methods used, design, data collection processes), what and how mental health was addressed (aspects of mental health addressed, theory or framework used to address mental health, mental health supports and interventions, outcome and evaluation), social determinants of health addressed (using definitions from Public Health Agency of Canada, 2024), and discussion sections of the literature (findings, limitations, implications, and future research). Descriptions of charting criteria are provided in Supplementary Materials File S4. Appendix A contains a charting table with some extracted information not addressed in Section 3 (Results). Any identified discrepancies were addressed through consensus between the three authors.

### 2.7. Analysis and Synthesis of Data

A qualitative content analysis was conducted on the extracted data pertaining to the findings on mental health support and interventions for SOGIE asylum seekers from the seven included documents. The qualitative content analysis process involved immersion in the data, development of codes and coding schemes, and developing categories by grouping codes together into meaningful clusters (Hsieh & Shannon, 2005). An inductive approach was used (Elo & Kyngäs, 2008), with a focus on manifest content (Kleinheksel et al., 2020). NVivo 15 software was used for coding (Lumivero, 2025).

As part of trustworthiness standards for content analysis, A.Y.-P.S. was immersed in the data throughout the literature review, screening, and extraction phases, which helped him better understand the research context of this topic. A.Y.-P.S. maintained a reflexive journal during the analysis process (Kleinheksel et al., 2020). As a result, A.Y.-P.S. worked to minimize his biases after becoming more mindful of how his lived experiences involving these shared identities and past experiences in mental health advocacy informed his interpretations. A.Y.-P.S. also created analytical memos to note his observations of patterns in the extracted data (Hsieh & Shannon, 2005). Lastly, S.Y. and T.J.C. reviewed the charting and analysis, which allowed for greater reflexivity and depth of understanding regarding the resulting categories (Elo & Kyngäs, 2008). During team meetings, S.Y. and T.J.C. critically asked A.Y.-P.S. to justify how interpretations were made in order to identify potential biases, and also provided feedback and input to arrive at consensus.

## 3. Results

### 3.1. General Summary of Results

Seven documents met the study’s inclusion criteria, which included four peer-reviewed articles (Abramovich et al., 2020; Kahn & Alessi, 2018; Lee & Brotman, 2013; Logie et al., 2016), and three grey literature sources (Hall & Sajnani, 2015; Mulé & Gates-Gasse, 2012; Sajnani, 2014). Of the four peer-reviewed articles, two were identified during the snowball searches with articles at the full-text screening stage (Abramovich et al., 2020; Lee & Brotman, 2013). All grey literature was output of the Envisioning Global LGBT Human Rights partnership, which is based at York University in Toronto, Ontario (Envisioning Global LGBT Human Rights, n.d.). Six were published between 2012 and 2017, and one was published in 2020. All documents had most of their participants from or were based in Ontario or Quebec. Only one article had participants from Nova Scotia (two participants), British Columbia (one participant), and Manitoba (one participant; Kahn & Alessi, 2018).

All included documents used qualitative designs, including community partnerships and participatory action research (Lee & Brotman, 2013; Logie et al., 2016; Mulé & Gates-Gasse, 2012), grounded theory (Kahn & Alessi, 2018; Lee & Brotman, 2013), narrative thematic analysis (Logie et al., 2016), and a case study (Abramovich et al., 2020), with two documents (Hall & Sajnani, 2015; Sajnani, 2014) not describing the design or analysis. Data collection processes involved interviews (Kahn & Alessi, 2018; Lee & Brotman, 2013; Sajnani, 2014), focus groups (Hall & Sajnani, 2015; Logie et al., 2016), patient records (Abramovich et al., 2020), and roundtable discussions (Mulé & Gates-Gasse, 2012).

### 3.2. Participant Demographics

The majority of the peer-reviewed documents had participants who were asylum seekers, refugee claimants, and other immigration statuses and identities combined (e.g., forced migrants, refugees, permanent residents, visa holders, non-status, service providers, advocates). Articles with multiple immigration statuses were included when it was possible to distinguish the unique experiences of SOGIE asylum seekers. The grey literature included (Hall & Sajnani, 2015; Mulé & Gates-Gasse, 2012; Sajnani, 2014) did not report participant demographics (e.g., number of participants, age, country of origin, gender identity, and sexual orientation) of the SOGIE asylum seekers and refugee claimants. Therefore, the following three paragraphs represent the included peer-reviewed articles (*n* = 4).

The number of participants in the studies ranged from one to twenty-nine. Based on Statistics Canada’s age categories (youth: 15–24 years old; adult: 25–64 years old; Statistics Canada, 2024), one article focused on youth (Abramovich et al., 2020), one article focused on youth and adults (Kahn & Alessi, 2018), one article focused on adults (Logie et al., 2016), and one article did not report the age of participants (Lee & Brotman, 2013).

Participants’ countries of origin varied in specificity between reporting countries and regions. This included the Eastern Mediterranean region (Abramovich et al., 2020; Kahn & Alessi, 2018; Lee & Brotman, 2013), the African region (Kahn & Alessi, 2018; Lee & Brotman, 2013; Logie et al., 2016), the Caribbean region (Kahn & Alessi, 2018; Lee & Brotman, 2013; Logie et al., 2016), the Southeast Asian region (Kahn & Alessi, 2018), Asia (unspecified; Lee & Brotman, 2013) and Latin America (Lee & Brotman, 2013).

Most of the participants in the study were cisgender men and women (Lee & Brotman, 2013; Logie et al., 2016), with few gender non-conforming (Lee & Brotman, 2013) and trans participants (Abramovich et al., 2020; Kahn & Alessi, 2018; Lee & Brotman, 2013; Logie et al., 2016). Participants’ sexual orientation was not reported at times (Abramovich et al., 2020; Lee & Brotman, 2013), but when reported, participants were bisexual (Logie et al., 2016), gay (Kahn & Alessi, 2018; Logie et al., 2016), and lesbian (Kahn & Alessi, 2018; Logie et al., 2016). The categorizations reported in all documents were primarily based on Western conceptualizations of sexual orientation and gender identity.

### 3.3. Identified Mental Health Burdens

Although this scoping review focused on strengths, numerous mental health burdens were identified throughout the included literature to contextualize mental health needs and individual participant strengths. Discrimination was a mental health burden identified in all included literature. Other identified mental health burdens include trauma, depression, suicidality, transphobia, stigma, anxiety, social isolation, homophobia, PTSD, substance use, chronic stress, and gender identity. Table 2 indicates which mental health burden was addressed within the included literature.

### 3.4. Identified Mental Health Needs and Strengths

The reporting of strengths-based aspects of participants’ mental health was varied across six of the seven included documents. Five described interpersonal support in the form of social connection (Abramovich et al., 2020; Hall & Sajnani, 2015; Logie et al., 2016; Mulé & Gates-Gasse, 2012) or a sense of belonging (Lee & Brotman, 2013; Logie et al., 2016) as strengths. One document (Kahn & Alessi, 2018) described how SOGIE refugee claimants may take a break from services and need time to reconnect with themselves after receiving their refugee status. They implied that this break was important for service providers to understand SOGIE refugee claimants’ well-being after the intensity of the asylum-seeking process (Kahn & Alessi, 2018). Two articles addressed the importance of self-affirmation for SOGIE asylum seekers’ mental health, in the general context of SOGIE identity (Logie et al., 2016), and in the context of trans identity (Abramovich et al., 2020).

### 3.5. Theory or Framework Describing Mental Health Support

Many of the peer-reviewed and grey literature conceptualized or described mental health support within the framework of intersectionality (Hall & Sajnani, 2015; Lee & Brotman, 2013; Logie et al., 2016; Mulé & Gates-Gasse, 2012; Sajnani, 2014) and social determinants of health (Abramovich et al., 2020; Kahn & Alessi, 2018; Logie et al., 2016; Mulé & Gates-Gasse, 2012). Other theories used include queer, trans, and critical race theory (Lee & Brotman, 2013), along with the minority stress model and a social ecological approach (Logie et al., 2016).

### 3.6. Programme Evaluation or Outcome Measurement of Mental Health Supports

No formal programme or outcome evaluation was identified or reported for the mental health supports provided. However, qualitative reporting of outcomes of mental health supports was provided in some included literature. For example, Abramovich et al. (2020) included descriptions of outcomes in addressing various social determinants of health for a single case of a trans refugee claimant. Likewise, Logie et al. (2016) noted some outcomes from participating in a social support group, which included participants describing an increased sense of social and emotional support and decreased stress about the immigration process.

### 3.7. Qualitative Content Analysis

**Figure 2:**
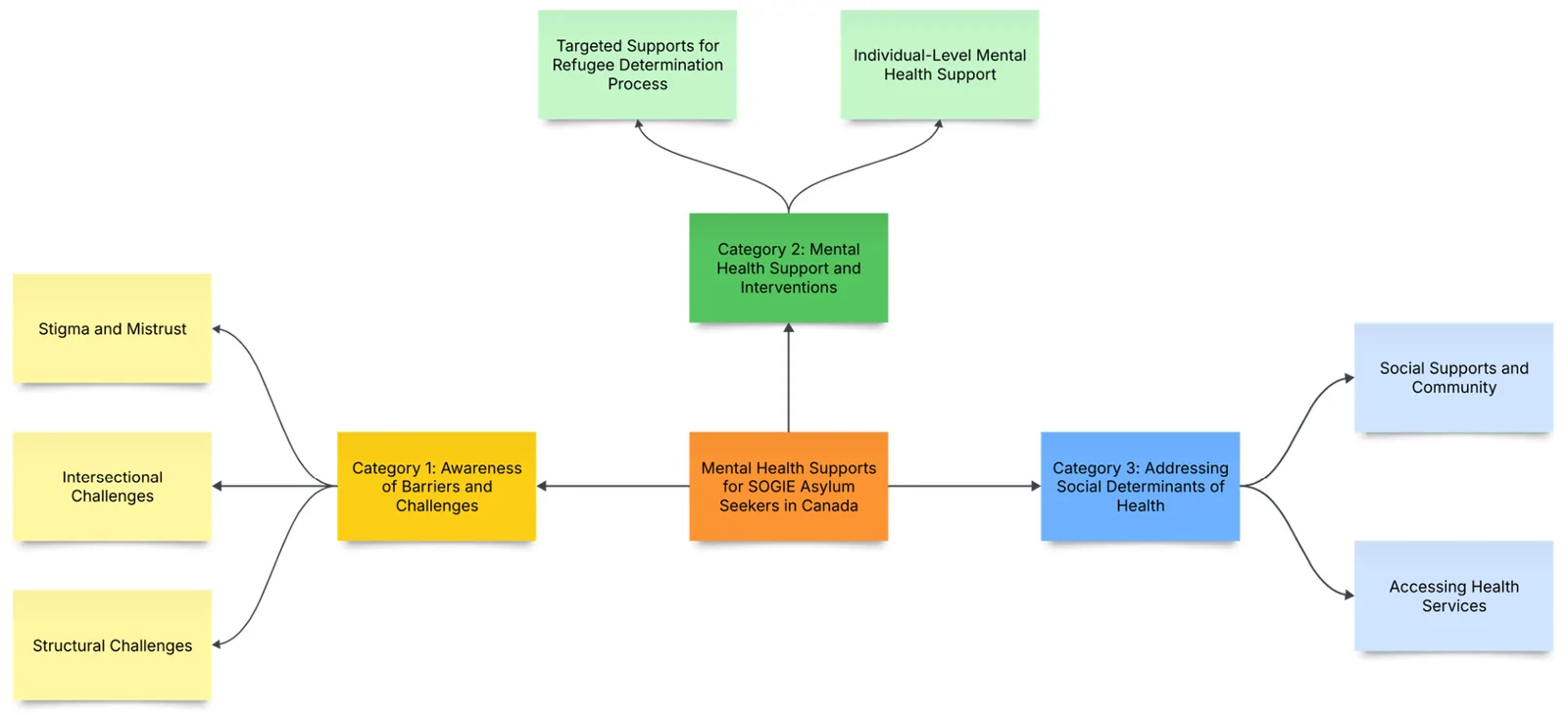
Conceptual map of qualitative content analysis findings. Note. The figure was created using Lucid (lucid.co).

Three key categories were identified regarding SOGIE asylum seeker mental health support from the included literature (Figure 2): awareness of barriers and challenges; mental health support and interventions; and addressing social determinants of health. Table 3 identifies the included literature that was associated with each sub-category.

#### 3.7.1. Awareness of Barriers and Challenges

The first category describes the reported barriers and challenges that were noted in the literature as considerations when providing mental health support to SOGIE asylum seekers in Canada. This includes the subcategories of stigma and mistrust, intersectional challenges, and structural challenges.

Stigma and mistrust were identified as potential barriers to reaching SOGIE asylum seekers and providing mental health support. Given that individuals may not have discussed mental health openly throughout their life and migration journey, SOGIE asylum seekers may find it challenging to connect with services for their mental health, as noted by one author: “This stigma can result in a reluctance to seek out mental health services, as many newcomers’ only concept of mental health services is psychiatrists and medication; sitting down and talking with a stranger can feel very unfamiliar” (Sajnani, 2014, p. 32). This account suggests that pre-migration experiences impact how SOGIE asylum seekers access mental health services in Canada. This reluctance may come from the criminalization of mental illness and SOGIE identity or exposure to a medicalized model of mental health services in the country of origin. Additionally, there may be mistrust in accessing supports, as SOGIE asylum seekers may not trust spaces that are directly promoted for SOGIE individuals (Hall & Sajnani, 2015). This also suggests that SOGIE asylum seekers may have had previous negative interactions when seeking help for their identity, and the sense of mistrust continues post-migration.

Intersectional challenges in providing mental health support to SOGIE asylum seekers were also noted. This involved various identity factors, such as culture and language: “She was referred to a transgender community-based drop-in program to encourage connection to community, but the language and cultural barriers prevented her from feeling that she belonged.” (Abramovich et al., 2020, p. E10). These authors highlight how the lack of language-, racial-, or ethnic-specific services has the potential to limit access to services for SOGIE asylum seekers who are members of racialized communities. Geography was another barrier that was noted, as SOGIE individuals located in rural or remote communities faced the additional barrier of accessing mental health services (Sajnani, 2014). When considering the barriers SOGIE asylum seekers already face at the intersection of gender identity, sexual orientation, and migration status, the available literature indicates the importance of addressing intersectional challenges when providing support for SOGIE asylum seekers.

Lastly, structural barriers related to the immigration and determination process were identified. SOGIE asylum seekers experienced barriers in accessing mental health services:

*“In reality, there is often no time for mental health care, or truly any health care, before an individual’s hearing. By the time they get to a care provider, it is often too late to obtain mental health evidence to support a claim”.*

(Sajnani, 2014, p. 32)

This author highlights how the time-limited structure of the refugee determination process creates major challenges in SOGIE asylum seekers accessing necessary and timely support. In addition, government funding and general limitations of accessible mental health supports were noted in the literature as barriers to providing adequate mental health support to SOGIE asylum seekers (Mulé & Gates-Gasse, 2012). These accounts suggest the need for specialized services that recognize the complex and nuanced needs of SOGIE asylum seekers and meet the increasing demand for these services.

#### 3.7.2. Mental Health Support and Interventions

The second category describes the mental health supports and interventions identified for SOGIE asylum seekers within a Canadian context. The first subcategory identifies specific mental health supports for SOGIE asylum seekers during the refugee determination process that can help ease the mental health burdens and challenges. This was seen when previous SOGIE refugee claimants who have successfully gone through the refugee hearing process can be a source of emotional support and encouragement (Logie et al., 2016). In addition to practitioners providing emotional support, this participant provides insight into how former refugee claimants can emotionally support SOGIE refugee claimants. Providing clarity on the refugee determination process was also highlighted as an important intervention:

*“[Among Friends, an LGBT Refugee Claimants Group] offer[s] workshops and information sessions on different things. Most are about Canada, what’s expected, what are your rights and responsibilities as an LGBT refugee claimant. We also help them with their hearing, which is very important to an LGBT refugee claimant”.*

(Mulé & Gates-Gasse, 2012, p. 27)

This description from the available literature highlights how community organizations can play an important role in providing SOGIE refugee claimants the information they need to understand and feel prepared in this time-limited and stressful determination process.

Another subcategory addressed one-to-one psychological interventions to support SOGIE asylum seeker mental health, such as counselling and psycho-diagnostic work. In their case study, Abramovich and colleagues explained how the trans refugee claimant patient received psycho-diagnostic support to identify a PTSD diagnosis alongside concurrent treatment for gender dysphoria (Abramovich et al., 2020). The authors describe how psychological support for SOGIE refugee claimants should address the mental health burdens identified in this population, such as gender dysphoria and trauma. In addition, the account also recognized the importance of psychological assessment, as the severity of trauma and other potential mental health burdens may not have been initially identified. Lee and Brotman (2013) describe another intervention where practitioners can connect individual experience with structural issues to address internalized oppression. This account notes how a service provider can work with queer and trans refugee claimants to reduce the burden on the individual by highlighting how different systems of power have, and continue to impact, their migration experience. A final intervention, and important consideration for service providers and organizations, is how SOGIE refugee claimants may choose to distance themselves from others and organizations once they receive a positive hearing decision and are legally recognized as refugees to “… reconnect with themselves, to live their life however they want to” (Kahn & Alessi, 2018, p. 33). This account describes how SOGIE refugee claimants, once successful, may need time to be with themselves to heal and process their migration journey. This likely includes processing the refugee determination process itself, alongside their experiences during pre-migration and while in transit.

#### 3.7.3. Addressing Social Determinants of Health

The third category highlights the importance of addressing the needs of SOGIE asylum seekers in a holistic manner. Two social determinants of health that were highlighted within the available literature were social support and access to health services.

Social support and community were noted when providing support for SOGIE asylum seekers, particularly as part of monthly programming that provides social spaces for support, such as the Foreign Integration programme provided by the Black Coalition for AIDS prevention (Mulé & Gates-Gasse, 2012). This description highlights how components of social support can be incorporated alongside community programming that is focused on providing information and knowledge about integration. As a result of having spaces for social connection, interpersonal relationships extend into community settings and are then strengthened and maintained (Logie et al., 2016). In doing so, SOGIE refugee claimants have an opportunity to develop a sense of community and support throughout their refugee determination process and in everyday challenges and successes.

Another social determinant of health identified was access to health services. This involved providing education to SOGIE refugee claimants about how to navigate Canada’s health care system and seek medical supports (e.g., how benefits work, specific terminology, the purpose of health insurance; Logie et al., 2016). This suggests the importance for service providers and organizations to provide adequate information and education about the Canadian health care system to ensure SOGIE refugee claimants can access the medical care they require. For trans individuals, access to medical care is even more crucial. Within the included case study (Abramovich et al., 2020), accessing gender-affirming care to address gender dysphoria was noted. After the patient completed physical exams and blood tests and experienced clinical benefits from hormone therapy, she was referred for vaginoplasty to address continued experiences of gender dysphoria (Abramovich et al., 2020). This account describes how trans refugee claimants may need a range of medical services, referrals, and coordination of care to provide adequate and appropriate gender-affirming care.

## 4. Discussion

This scoping review aimed to identify existing approaches to support the mental health of SOGIE asylum seekers within Canada based on the findings from four peer-reviewed studies and three grey literature documents. Alongside addressing social determinants of health, emotional and psychological support during the refugee determination process was indicated as important for supporting SOGIE asylum seekers’ mental health. Findings also noted the importance of being aware of how stigma, mistrust, intersectional challenges, and structural barriers can impact how mental health supports are provided to and received by SOGIE asylum seekers in Canada. Given the limited available literature regarding this topic, interpretations will be based upon the findings from the included literature alongside extant literature on refugee, asylum seekers, and SOGIE mental health from Canada and around the world.

### 4.1. Interventions and Approaches for Mental Health Support

The findings of this scoping review highlighted the importance for SOGIE asylum seekers to receive psychological interventions and informational support throughout the refugee determination process. These findings are consistent with how practitioners in other countries have continued to work with SOGIE refugee and asylum seekers in managing mental health concerns that were associated with their pre-, in-transit, and post-migration journey (Hermaszewska et al., 2022; Piwowarczyk et al., 2017). Within existing literature, this can include how providers working with sexual minority refugees and asylum seekers provide psychoeducation and therapy to address trauma (Alessi, 2016; Bird et al., 2024) and equip clients with skills to address the continued experiences of stigmatization and racism (Bird et al., 2024). Alessi and Kahn (2017) have developed a clinical practice framework when working with SOGIE asylum seekers, which includes providing psychological support during the determination process. Therapists can support SOGIE asylum seekers to communicate their trauma narrative for the determination within a human rights lens, teach clients grounding and mindfulness techniques to cope with stress and reactivation of trauma symptoms, and explain how identity labels are used and differ from those in their country of origin (Alessi & Kahn, 2017).

SOGIE asylum seekers may also benefit from receiving education and information about Canada’s refugee determination process. Based on the reviewed literature, education and advocacy from practitioners and community organizations can have a role in supporting SOGIE asylum seekers in their claims process. This includes providing SOGIE asylum seekers with information about the determination process and support for the hearing (Mulé & Gates-Gasse, 2012). Since SOGIE asylum seekers and refugees can experience challenges in accessing information and resources related to legal services (Mulé, 2022), providing clarity about the process can be one way to help reduce the mental health burdens that SOGIE asylum seekers experience during this stressful period.

A novel finding highlighted by this scoping review is that SOGIE refugee claimants who have received a positive hearing decision may choose to distance themselves from the services they have accessed throughout their determination process (Kahn & Alessi, 2018). Existing research focuses on the extent of mental health and health care utilization among refugees and asylum seekers (Mazumdar et al., 2022; Satinsky et al., 2019), which is crucial for developing interventions, programming, and services and for effectively addressing existing barriers to access. However, the focus on utilization may not recognize the nuances associated with post-hearing lived experiences of SOGIE individuals. Given that the refugee determination process is time-bound (Sajnani, 2014), involves multiple re-tellings of vulnerable and traumatic life experiences (Gottvall et al., 2023), and requires navigation of novel and complex systems with existing mental health burdens from the migration journey (Alessi et al., 2021), immense burdens, stress, and likely fatigue may have been experienced. The decision for SOGIE asylum seekers post-hearing to take a break from services could act as an opportunity for them to determine the pace with which they engage with their healing on their own terms. This finding creates awareness for service providers and organizations to understand the lived experiences of SOGIE asylum seekers post-hearing, which informs how mental health services are developed and implemented.

With regard to specific approaches, an intersectional framework was often noted across the literature reviewed in this scoping review. Originally named and defined by Kimberlé Crenshaw, intersectionality addresses how different identity factors intersect and interact with systems of power in society, and how these experiences of marginalization have been shaped and normalized by these power structures (Carbado et al., 2013). The goal of intersectionality is to highlight the dynamics that perpetuate experiences of marginalization and generate social change within structures (Carbado et al., 2013). Intersectionality is relevant for understanding SOGIE asylum seeker and refugee experiences, as their experiences of oppression are shaped by how migration and sexuality are conceptualized socially, politically, historically, culturally, and more through existing policies and procedures (Brotman & Lee, 2011). By having this framework, practitioners can then provide more competent care that meets the intersectional needs of SOGIE asylum seekers across their various identities (Alessi & Kahn, 2017), such as gender identity, sexual orientation, migration status, ethnicity, and more. Examples of the need for an intersectional approach with SOGIE refugees and asylum seekers have been documented within existing literature (Hermaszewska et al., 2022; Lee & Brotman, 2011). These findings from this review and from extant literature indicate how the intersectionality framework is important when providing psychological support to SOGIE asylum seekers.

### 4.2. Addressing Social Determinants of Health Alongside Mental Health Supports

Social determinants of health were often addressed alongside mental health care for SOGIE asylum seekers within this scoping review. Alongside time pressures associated with the refugee determination process (Sajnani, 2014), SOGIE asylum seekers experience a range of challenges such as housing instability, social isolation, unemployment, and financial challenges, which create additional challenges and struggles (Abramovich et al., 2020; Hall & Sajnani, 2015). This suggests that a holistic and timely approach to addressing the needs of SOGIE asylum seekers is crucial. Existing research conducted by Murugan et al. (2024) also noted the importance of addressing social determinants of health when providing care for SOGIE refugees. Additionally, the application of the social determinants of health framework has been used with other refugee and asylum seeker communities to understand health impacts and to provide appropriate and timely interventions and support (Kuo et al., 2020; Oren et al., 2026). A recommendation by Kuo et al. (2020) was the importance of community collaboration across multiple disciplines to appropriately support the needs of refugee newcomers, which was also identified as part of this scoping review with SOGIE asylum seekers.

Social support was noted as an important social determinant of health to address among SOGIE asylum seekers, which involves facilitating social support and a sense of community with other SOGIE asylum seekers and refugees through programming and services. An important consideration is that not all social support may be helpful, as SOGIE refugees and asylum seekers often experience social exclusion within host countries from their diaspora communities and from members of the host country (Gottvall et al., 2023; Lee & Brotman, 2011). Therefore, SOGIE asylum seekers should access social support from sources that are affirming of one’s identities and are culturally responsive (Alessi & Kahn, 2017). This can involve connecting with service providers and community organizations that are affirming of LGBTQ+ identities and have a cultural focus (Gottvall et al., 2023; Lee & Brotman, 2011; Nematy et al., 2023). Another crucial source of social support can come from other SOGIE asylum seekers and refugees, as identified in existing literature. Fox et al. (2020) noted that SOGIE asylum seekers were keen to connect with other SOGIE asylum seekers and to provide mentorship to new arrivals. Alessi (2016) noted that participants found it helpful to have friends who had completed the asylum or refugee process validate the hardships and frustrations associated with the process. In addition, participants who had gone through the asylum or refugee process also wanted to give back to those who were going through that process (Alessi, 2016). This suggests that SOGIE asylum seekers and refugees may have a reciprocal way of receiving support and a desire to give back to the next group of arrivals. However, some SOGIE asylum seekers may not feel ready to connect with others and must have the opportunity to determine how things progress at their own pace (Alessi & Kahn, 2017).

Improving access to health services for SOGIE asylum seekers was another social determinant of health highlighted within this scoping review. This can involve providing education about the Canadian health care system, such as the system entry points and ways to navigate the health care system, along with providing access to gender-affirming care for trans asylum seekers. Existing research has highlighted that barriers exist for refugee and asylum seekers in accessing health services, such as language barriers and lack of culturally competent care (Asgary & Segar, 2011; McKeary & Newbold, 2010). However, Kuo et al. (2020) noted how satisfaction with health services was positively associated with mental health, which speaks to how interactions with health care providers can play a role in mental health. Therefore, by providing interventions such as cultural mediators, language interpretation services, and culturally relevant care, accessibility to and experience of primary health care services for refugee and asylum seekers can be improved (Iqbal et al., 2022). Existing literature has also noted how gender-affirming health care, such as hormone therapy and gender-affirming surgery, may be associated with improved health and quality of life for trans individuals (Baker et al., 2021; Pinna et al., 2022). For trans forced migrants, they can access a range of health care services for their needs, such as community clinics, medications, and gender care (Hermaszewska et al., 2022). Organizations may be aided by having a network of community referrals to support coordinated care to provide these services for trans asylum seekers, as what was demonstrated by Abramovich et al. (2020).

### 4.3. Addressing Barriers and Challenges

Even though this scoping review focused on identifying mental health supports for SOGIE asylum seekers in Canada, a brief exploration of the barriers and challenges identified is necessary to understand the full context of their mental health-related experiences. Based on the available literature included, the barriers and challenges in providing mental health support to SOGIE asylum seekers in Canada involved stigma and mistrust, intersectional challenges, and structural barriers.

Based on accounts from this review, SOGIE asylum seekers may be hesitant to talk about their mental health and have a mistrust of spaces that are promoted for SOGIE individuals. With regard to stigma towards mental health, the experiences SOGIE asylum seekers had in their country of origin may play a role. Faleti and Akinlotan (2025) conducted a systematic review and identified how mental illness stigma within African countries exists across the systems of family, community, and health care, and at the levels of the self, public, associative, and institutional. As a result, individuals are dehumanized, experience abuse, and become hesitant to seek support (Faleti & Akinlotan, 2025). With regard to mistrust of SOGIE spaces, findings from this review and existing literature have documented the extensive identity-based and state-sanctioned violence and discrimination experienced by SOGIE asylum seekers during pre-migration and while in transit (Alessi et al., 2021; Yarwood et al., 2022). As a result, SOGIE asylum seekers and refugees can be fearful and mistrustful of others or may not be comfortable disclosing their identity (Alessi, 2016; Bird et al., 2024), which can be carried into interactions with service providers and community organizations in the Canadian context. These findings within this scoping review and existing literature suggest that it is important to understand how pre-migration and in-transit experiences of stigma and mistrust inform how SOGIE asylum seekers present themselves when accessing services in the Canadian context.

This scoping review also highlighted how intersectional challenges exist that prevent SOGIE asylum seekers from accessing the mental health support they need. This included barriers regarding culture and language (Abramovich et al., 2020), along with geography (Sajnani, 2014). These findings are consistent with existing research that identifies a lack of intersectional support for SOGIE asylum seekers and refugees. Examples include SOGIE refugees and asylum seekers unable to access services that were culturally appropriate, linguistically relevant, and affirming of one’s gender identity and sexual orientation (Hermaszewska et al., 2022; Kahn et al., 2018; Murugan et al., 2024; Nematy et al., 2023). This indicates how intersectional challenges regarding support for SOGIE asylum seeker mental health have been continuously identified, which speaks to the need for greater intersectional support.

Consistent with findings from previous scoping reviews (e.g., see Lee et al., 2021), structural challenges were also identified as a barrier in providing mental health supports for SOGIE asylum seekers in Canada in the form of compressed timelines in the determination process and the limited or conditional government funding provided to community organizations. Even though these findings were based on the early-to-mid 2010s (Mulé & Gates-Gasse, 2012; Sajnani, 2014), Canada has continued to create structural barriers that limit the ability to provide appropriate and timely mental health support. This includes the passing of Bill C-12—Strengthening Canada’s Immigration System and Borders Act into law in March 2026 (IRCC, 2026a), which creates additional risks and vulnerabilities for SOGIE asylum seekers (Canadian Council for Refugees, 2025). These structural barriers have also been identified in other countries, such as Australia, which has highlighted how the asylum system and the lack of safe and supportive spaces for SOGIE asylum seekers and refugees is itself a form of structural violence (Istiko et al., 2024).

### 4.4. Study Characteristics

The included literature in this scoping review was predominantly based in the provinces of Ontario and Quebec, as compared to an anticipated equal distribution of research across Canada. With the majority of asylum claims being submitted in the province of Ontario or Quebec from March 2023 to March 2026 (IRCC, 2026b), this seems to be understandable. However, British Columbia and Alberta were also identified as the provinces with the third and fourth highest number of asylum claim submissions (IRCC, 2026b), with minimal to no published research on SOGIE asylum seeker mental health in these provinces. Despite this, community-level organizations for SOGIE asylum seekers do exist across Canada. This includes DIVERSEcity, MOSAIC, and the Rainbow Refugee Society in British Columbia; the Rainbow Refuge Program and End of the Rainbow Foundation in Alberta; the 519 and Access Alliance Multicultural Health and Community Services in Ontario; AGIR Montréal in Quebec; and the Rainbow Refugee Association of Nova Scotia. This suggests how grassroots and community organizations are responding as best they can to the needs of SOGIE asylum seekers across Canada, despite the regional concentration of research literature in Ontario and Quebec.

Another observed pattern within the included literature was how the demographics for SOGIE asylum seekers were often reported in combination with participants of other immigration statuses (e.g., refugees) or were either partially or fully undisclosed. One potential consideration is that participants themselves may not disclose certain demographic information. Some reasons can include having a different understanding of sexual orientation and gender identity that is not conceptualized within Western frameworks, grappling with the cultural appropriateness of identity disclosure, discomfort with expectations of identity-based disclosure within Western spaces, and worries about potential discrimination (D’souza et al., 2022; Lee et al., 2021). Another consideration of the inconsistent reporting of participant demographics may reflect the ethical considerations that researchers have in protecting SOGIE participant privacy and safety, particularly in smaller communities. This aligns with fears that SOGIE asylum seekers have of people back in their country of origin finding out about their identity and increasing risks of victimization (Alessi et al., 2021). Simultaneously, there is a crucial need for research that is intersectional, embraces complexity, and provides greater opportunities to critically analyze existing power systems that maintain and perpetuate inequities and marginalizations (Bowleg et al., 2023). SOGIE asylum seekers are a group of individuals who are intersectionally invisible within the broader SOGIE newcomer context. Despite sharing similar intersectional marginalizations and inequities based upon gender identity, sexual orientation, race, and ethnicity, SOGIE asylum seekers experience inequities and burdens from the Canadian refugee determination process that SOGIE refugees may not experience. SOGIE asylum seekers have greater precarity and decreased access to many essential services, while simultaneously experiencing re-traumatization from re-telling their experiences of trauma as part of preparing their asylum narrative. This observation highlights the balance that researchers have in ensuring that participant identities are protected, while also reporting demographics of SOGIE asylum seekers sufficiently. In doing so, this can support the development of appropriate interventions and research agendas for SOGIE asylum seekers, alongside efforts that support SOGIE refugees and newcomers.

### 4.5. Limited Evidence Base for Effective Mental Health Interventions

A tension that occurred during this scoping review was balancing the importance of providing practical recommendations while simultaneously navigating the limited evidence base that exists for SOGIE asylum seeker mental health research in Canada. Based on the very little evidence that exists, only tentative recommendations and interpretations could be made regarding potential components that could support SOGIE asylum seeker mental health. However, given that scoping reviews help inform researchers about the state of research in this area, this lack of evidence base itself speaks to the need for further research into this area regarding SOGIE asylum seeker mental health supports within the Canadian context.

The limited evidence base also suggests an additional inequity that SOGIE asylum seekers experience specifically within the realm of research. Alongside the intersectional challenges and structural barriers that they experience when accessing and receiving mental health services, SOGIE asylum seekers in Canada are also experiencing structural inequities in research evidence for effective mental health interventions. This was noted in the absence of any formal programme evaluation or outcome measurement for mental health interventions in Canada for this population, which creates major gaps in knowing what works and tangible impacts. The absence of findings for specific mental health interventions coincided with the questions we received from our colleagues at ENC, as they also noted challenges in finding research evidence about this topic. As a result, this major gap in this research area does impact the quality of evidence-based interventions that can be provided along with policy development for SOGIE asylum seekers within Canada.

### 4.6. Limitations

There were a few limitations for the scoping review concerning extant literature, geographical representativeness of the included literature, and reported demographic variables of SOGIE asylum seekers that limit the generalizability of these findings. There were a limited number of peer-reviewed articles and grey literature included within the scoping review, which may reflect limited research into mental health supports for SOGIE asylum seekers across Canada. From this limited sample of literature, all included peer-reviewed studies and grey literature were qualitative, which limits any statistical understanding or application of SOGIE asylum seekers’ experiences related to mental health supports. The included literature has a geographic concentration in Ontario and Quebec, which limits generalizability of the findings to other Canadian provinces and territories. Additionally, the findings of this scoping review were based predominantly on cisgender men and women, which limits the generalizability of the findings to the mental health supports that are needed for trans and non-binary asylum seekers in Canada. Documents also needed to be written in English for inclusion, which limits understanding of interventions and mental health supports for this population in other languages (e.g., exclusion of French-language documents).

Given the small sample of literature on this topic yielding seven documents, charting, extraction, and analysis were completed by only one extractor and synthesist. Due to the small sample size, charting was discussed with the research team to minimize biases. Additional measures were taken to minimize bias, which included reflexive journalling, statements of researcher positionality, continued dialogue between authors regarding findings, and the integration of feedback into the extraction and interpretation of results.

### 4.7. Implications

#### 4.7.1. Practical Implications

SOGIE asylum seekers can come from many countries around the world, which indicates that needs can vary and psychological support must be tailored to directly address the concerns that are identified. As such, practitioners and organizations should conduct a needs assessment to identify the needs associated with SOGIE asylum seekers within their region. From there, practitioners and organizations should commit to seeking funding and develop programmes that are tailored to address the identified needs from the needs assessment. Afterwards, engaging in programme evaluation once support and care are completed is essential in understanding what components of the programme were helpful and what can be adjusted for better outcomes. Depending upon the sequencing and services offered, publishing the needs assessment and programme evaluation data mobilizes knowledge for researchers and practitioners across all provinces and territories in Canada. In doing so, this work addresses the need for more programme evaluation of mental health services for SOGIE asylum seekers, may generate greater attention for researchers, and can support practitioners across Canada to build upon knowledge collectively and improve support for this population.

Practitioners and organizations that support SOGIE asylum seekers should provide psychological support that uses an intersectional framework to best understand how power structures within Canadian society play a role in their experiences of inequity and marginalization. In line with an intersectional framework, services should be affirming of SOGIE identities, cultural identity, and migration status. Services must also center safety and sensitivity to proactively address the stigma and mistrust that SOGIE asylum seekers may have when accessing services. With SOGIE asylum seekers often experiencing trauma and other mental health burdens throughout the migration journey, establishing safety is key (Alessi & Kahn, 2017). Practitioners and organizations also play a crucial role in providing SOGIE asylum seekers clarity and support as part of the refugee determination process, such as explaining their rights, what services they have access to, and anticipated timelines. Practitioners and organizations also should be mindful that SOGIE refugee claimants may distance themselves from services after receiving a successful asylum hearing decision, which may, in part, be a way to determine their own pace of navigating through experiences.

In conjunction with psychological support, social determinants of health must be addressed to ensure the various and immediate needs of SOGIE asylum seekers are met when in Canada. This includes providing social support by facilitating spaces for SOGIE asylum seekers to develop social connections and community, and providing mentorship opportunities with other SOGIE asylum seekers and refugees. In addition, practitioners and service providers should provide education and support for SOGIE asylum seekers on how to engage with the Canadian health care system, along with facilitating medical referrals for gender-affirming care. Lastly, organizations and practitioners would benefit from having a network of community referrals to support coordinated care.

#### 4.7.2. Methodological Implications

This scoping review process demonstrated that community partners can be involved in shaping this research project. By having dialogue with community partners at Edmonton Newcomer Centre about their needs and how this project can support them with SOGIE asylum seekers, this strengthens relationships between researchers and community organizations and encourages knowledge mobilization. In addition, the inclusion of grey literature was also beneficial to ensure that findings from peer-reviewed research were supplemented and enhanced by grey literature to best respond to the needs of community partners.

#### 4.7.3. Research Implications

A major outcome of this scoping review highlights the need for more research on mental health supports and interventions for SOGIE asylum seekers within the Canadian context. This is based on the limited evidence base identified regarding mental health interventions for SOGIE asylum seekers in Canada, and that this topic has primarily been investigated within the context of the United States (Fox et al., 2026; Reading & Rubin, 2011). Given that the majority of SOGIE asylum seeker mental health research is currently based in the provinces of Ontario and Quebec, there is a need for researchers to investigate SOGIE asylum seeker mental health experiences in other provinces in Canada, such as British Columbia and Alberta. In addition, researchers who investigate SOGIE asylum seeker and refugee experiences should disaggregate the immigration statuses of their participants as best as possible while also protecting participant identity and safety. This will support researchers and other knowledge users in better distinguishing the support needed for SOGIE asylum seekers as they navigate migration experiences that include the refugee determination process. A greater research focus is also needed for the mental health supports of trans asylum seekers in Canada.

No formal programme evaluation or outcome measurement of mental health supports and interventions was identified. Moving forward, it would be beneficial to conduct programme evaluation research in collaboration with community organizations across Canada that currently provide support to SOGIE asylum seekers. In doing so, components of programming and mental health interventions that are effective for SOGIE asylum seekers within the Canadian context can be identified. Lastly, having more quantitative or mixed methods research methodologies would increase understanding about SOGIE asylum seeker mental health in Canada. Collaborating with existing community organizations across Canada that serve SOGIE asylum seekers may help bring together a greater number of SOGIE asylum seeker perspectives.

## 5. Conclusions

This community-responsive scoping review aimed to identify the approaches taken to support the mental health of SOGIE asylum seekers in Canada. The findings suggest psychological and informational support is helpful for SOGIE asylum seekers during the distressing and traumatic refugee determination processes. Mental health supports that are provided should use an intersectional framework. In addition, social determinants of health should be addressed alongside mental health supports, which can involve facilitating social supports and connection with other SOGIE asylum seekers and refugees, along with improving education and access to Canadian health care services and gender-affirming care. Research about SOGIE asylum seeker mental health must take place in more provinces outside of Ontario and Quebec, along with greater consideration for increased research collaborations with existing community organizations across Canada that support SOGIE asylum seekers.

## Figures and Tables

**Table 1:** MEDLINE search strategy in Population–Concept–Context (PCC) Framework.

PCC Framework	Query String
Population	(((sexual or gender) adj3 (orientat* or identi* or express* or divers*)) or SOGI* or SGM or GSM) OR (LGB* or GLBT* or LBG* or GLB* or 2SLGBT* or bisexual* or bi-sexual or bicurious* or bi-curious* or “gender* divers*” or gay? or homosexual* or lesbian* or lesbigay* or “men who have sex with men” or MSM or ((sexual* or gender* or romantic) adj3 minorit*) or non-heterosexual* or queer* or genderqueer* or “women who have sex with women” or WSW or two-spirit* or twin-spirit* or transsexual* or transgender* or transm?n or transwom?n or (trans adj2 (men or man or women or woman or boy or girl or person* or individual* or patient* or feminine or masculine)) or male-to-female or female-to-male or MTF or FTM or asexual* or aromantic* or non-binary or nonbinary or pansexual* or pan-sexual* or non-cisgender* or genderfluid* or gender-fluid* or genderless or agender or non-gendered or “third gender*” or bigender* or same-sex or “gender non conforming” or “gender non-conforming”) AND (asylum or asylee* or claim* or “force* migra*” or “undocumented migrant*” or exile* or precari* or “precari* status”) AND
Concept	(((mental or psych* or emotional or community or spirit*) adj3 (health* or well* or illness* or condition* or support* or diagnosis or resilien*)) or depress* or anxiety or “stress disorder*” or PTSD or post-traumatic stress disorder or post traumatic stress disorder or suicid* or “suicid* ideation” or trauma or alcohol misuse or substance misuse or treatment* or therap* or psychotherap* or counsel* or “psychosocial support” or “psychosocial intervention” or “psychosocial program*”) NOT (cancer or COPD or stroke or dialysis or diabetes or cardiovascular or parasit* or psoriasis or arthritis or vitiligo or pharma* or food or homeless* or “restless legs syndrome” or pregnancy or reproduction or tuberculosis or labor or labour or skeletal) AND
Context	(Canad* or “British Columbia” or “Colombie Britannique” or Alberta* or Saskatchewan or Manitoba* or Ontario or Quebec or “Nouveau Brunswick” or “New Brunswick” or “Nova Scotia” or “Nouvelle Ecosse” or “Prince Edward Island” or Newfoundland or Labrador or Nunavut or NWT or “Northwest Territories” or Yukon or Nunavik or Inuvialuit).

**Table 2:** Topics of mental health burdens identified in the included literature.

Mental Health Burden	Citation
Discrimination	(Abramovich et al., 2020; Hall & Sajnani, 2015; Kahn & Alessi, 2018; Lee & Brotman, 2013; Logie et al., 2016; Mulé & Gates-Gasse, 2012; Sajnani, 2014)
Trauma	(Abramovich et al., 2020; Hall & Sajnani, 2015; Kahn & Alessi, 2018; Lee & Brotman, 2013; Mulé & Gates-Gasse, 2012; Sajnani, 2014)
Depression and suicidality	(Abramovich et al., 2020; Hall & Sajnani, 2015; Kahn & Alessi, 2018; Mulé & Gates-Gasse, 2012; Sajnani, 2014)
Transphobia	(Abramovich et al., 2020; Hall & Sajnani, 2015; Lee & Brotman, 2013; Mulé & Gates-Gasse, 2012; Sajnani, 2014)
Stigma	(Abramovich et al., 2020; Hall & Sajnani, 2015; Logie et al., 2016; Mulé & Gates-Gasse, 2012; Sajnani, 2014)
Anxiety	(Abramovich et al., 2020; Hall & Sajnani, 2015; Kahn & Alessi, 2018; Mulé & Gates-Gasse, 2012; Sajnani, 2014)
Social isolation	(Abramovich et al., 2020; Hall & Sajnani, 2015; Mulé & Gates-Gasse, 2012; Sajnani, 2014)
Homophobia	(Hall & Sajnani, 2015; Lee & Brotman, 2013; Mulé & Gates-Gasse, 2012; Sajnani, 2014)
PTSD	(Abramovich et al., 2020; Hall & Sajnani, 2015; Kahn & Alessi, 2018)
Substance use	(Abramovich et al., 2020; Mulé & Gates-Gasse, 2012; Sajnani, 2014)
Chronic stress	(Hall & Sajnani, 2015; Kahn & Alessi, 2018; Mulé & Gates-Gasse, 2012)
Gender identity	(Abramovich et al., 2020; Lee & Brotman, 2013)

**Table 3:** Included literature associated with categories.

Category	Sub-Category	Associated Documents
Awareness of Barriers and Challenges	Stigma and Mistrust	(Hall & Sajnani, 2015; Mulé & Gates-Gasse, 2012; Sajnani, 2014)
	Intersectional Challenges	(Abramovich et al., 2020; Sajnani, 2014)
	Structural Challenges	(Abramovich et al., 2020; Mulé & Gates-Gasse, 2012; Sajnani, 2014)
Mental Health Support and Interventions	Targeted Supports for Refugee Determination Process	(Abramovich et al., 2020; Hall & Sajnani, 2015; Lee & Brotman, 2013; Logie et al., 2016; Mulé & Gates-Gasse, 2012)
	Individual-Level Mental Health Support	(Abramovich et al., 2020; Hall & Sajnani, 2015; Kahn & Alessi, 2018; Lee & Brotman, 2013; Mulé & Gates-Gasse, 2012)
Addressing Social Determinants of Health	Social Supports and Community	(Abramovich et al., 2020; Hall & Sajnani, 2015; Lee & Brotman, 2013; Logie et al., 2016; Mulé & Gates-Gasse, 2012)
	Accessing Health Services	(Abramovich et al., 2020; Logie et al., 2016; Mulé & Gates-Gasse, 2012)

## Data Availability

The original contributions presented in this study are included in the article/Supplementary Materials. Further inquiries can be directed to the corresponding author.

## Supplementary Materials

The following supporting information can be downloaded at: https://www.mdpi.com/article/10.3390/bs16091484/s1, File S1: Preferred Reporting Items for Systematic Reviews and Meta-Analyses extension for Scoping Reviews (PRISMA-ScR) Checklist; File S2: Search query strings; File S3: Grey Literature Search Plan Record; File S4: Entities to Chart Descriptions.

## Abbreviations

The following abbreviations are used in this manuscript:

IRB	Immigration and Refugee Board of Canada
IRCC	Immigration, Refugees and Citizenship Canada
JBI	Joanna Briggs Institute
LGBT	Lesbian, gay, bisexual, trans
LGBTQ	Lesbian, gay, bisexual, trans, queer
PCC	Population-Concept-Context
PRISMA-ScR	Preferred Reporting Items for Systematic Reviews and Meta-Analyses extension for Scoping Reviews
SOGIE	Sexual orientation, gender identity and expression
UNHCR	United Nations High Commissioner for Refugees

## Appendix A. Author, Year of Publication, Literature Type, Sample Size, Aim and Questions, and Main Findings Related to Mental Health Supports for SOGIE Asylum Seekers

**Authors,** **Year**	**Literature Type**	**Sample Size**	**Aim and Questions**	**Main Findings Related to Mental Health Supports for SOGIE Asylum Seekers**
(Abramovich et al., 2020)	Peer reviewed	1 (refugee claimant)	A clinical case study of a trans woman refugee claimant in Toronto, Canada, describing her pre- and post-migration experiences. Post-migration experiences include describing interactions with medical and mental health services and treatments, barriers, and various social determinants of health.	Described receiving psychotherapy and psychodiagnostic supports. Described access and connection with healthcare services (e.g., hormone therapy, referral for vaginoplasty), and clinical benefits experienced by the patient. Described connection with other services to support social determinants of health (e.g., language interpretation services, community-based social support, housing support, language support, and legal resources).
(Hall & Sajnani, 2015)	Grey literature	Not disclosed or applicable	An information sheet that describes the mental health and asylum experiences of LGBT asylum seekers in Canada, which is intended for the audience of Canadian service providers that work with LGBT asylum seekers.	Describing the importance of supportive environments to foster resiliency, self-efficacy, and feelings of shared identity. In addition, highlighted the importance of having access to resources that address their social, health, legal, and settlement needs. A consideration was shared regarding how services are promoted, as LGBT asylum seekers may not access services that are promoted as LGBT.
(Kahn & Alessi, 2018)	Peer reviewed	29 (service providers and LGBT forced migrants)	Exploring how service providers and LGBT forced migrants describe the psychological experiences of seeking refugee status in Canada, and the extent of psychological consequences post-claim.	Participant (an advocate) described how after going through the refugee claim process, individuals may step away from services and community events to reconnect with themselves and live on their own terms.
(Lee & Brotman, 2013)	Peer reviewed	28 (sexual minority refugees and refugee claimants, service providers, and advocates)	Using critical intersectionality analysis to analyze experiences of queer and trans refugees in Canada, and how findings can inform the work of social workers and community organizers that support queer and trans refugees.	Described interventions, such as accompaniment work, continued dialogue to support informed decision-making, and connecting individual experiences of the determination process to systemic barriers.
(Logie et al., 2016)	Peer reviewed	29 (refugees, permanent residents, visitor visa, student visa, non-status)	Exploring how a social support group provides benefits to LGBT African and Caribbean newcomers in Toronto, Canada.	Described how the social support group facilitated the development of friendships, improved emotional wellbeing, and created opportunities for other LGBT newcomers and community service providers to share knowledge about immigration processes and how to access the Canadian health care system.
(Mulé & Gates-Gasse, 2012)	Grey literature	Not applicable or disclosed	A document summarizing key takeaways and points from three events organized by the Canadian Team of Envisioning Global LGBT Human Rights in February and March 2012. The events included a roundtable discussion with community agencies about issues that LGBT asylum seekers are experiencing, a panel discussion about the impact of immigration reforms on LGBT asylum in Canada, and a panel discussion about how to support LGBT asylum seeker health and wellbeing.	Participants described the need for responsive mental health services (e.g., drop-in basis) for LGBT asylum seekers going through the claimant process and the use of an intersectional approach. Various sections in the document described other findings in relation to housing and poverty, settlement services, and challenges experienced during the determination process. The document also had a section that described services in Ontario that support LGBT asylum seekers. The Black Coalition for AIDS Prevention described providing monthly informational workshops for LGBT asylum seekers about the determination process and other settlement needs, along with a monthly social support and discussion group. The 519 runs the Among Friends program, which is an LGBT refugee claimants’ group that provides workshops and information sessions about the determination process. The 519 also provides 1:1 support, counselling, and legal advice in preparation for a hearing.
(Sajnani, 2014)	Grey literature	Not applicable or disclosed	Report that described impacts of the implementation of Bill C-31 in relation to LGBT asylum seekers in Canada. This included addressing Canada’s international obligations to support refugees, Canadian jurisprudence of LGBT asylum seekers, and the impact upon LGBT asylum seekers because of these changes.	Described summary points from interviews with mental health professionals in Ontario that support LGBT asylum seekers. Noted the shorter timelines, limited mental health services, and challenges with providing mental health services given these shorter timelines. In addition, barriers such as stigma, geography, language barriers, and financial coverage were discussed.
