# Supplementary material for: Mental Health Supports for SOGIE Asylum Seekers in Canada: A Community-Responsive Scoping Review
Source: Behav Sci (Basel). 2026 Aug 25;16(9):1484. doi: 10.3390/bs16091484 (PMC13603833; doi:10.3390/bs16091484)
Supplement: Supplementary file 1 [file behavsci-16-01484-s001.zip › behavsci-4379408-supplementary.pdf]

Supplementary Materials File for: Mental health supports for SOGIE asylum seekers in Canada: A community-responsive scoping review

Table of Contents

Supplementary Materials File S1) Preferred Reporting Items for Systematic reviews and Meta-Analyses extension for Scoping Reviews (PRISMA-ScR) Checklist 2

Supplementary Materials File S2) Search Query Strings 4

Supplementary Materials File S3) Grey Literature Search Plan Record 9

Supplementary Materials File S4) Entities to Chart Descriptions 43

Supplementary Materials File S1) Preferred Reporting Items for Systematic reviews and Meta-Analyses extension for Scoping Reviews (PRISMA-ScR) Checklist

SECTION	ITEM	PRISMA-ScR CHECKLIST ITEM	REPORTED ON PAGE #
**TITLE**			
Title	1	Identify the report as a scoping review.	p. 1
**ABSTRACT**			
Structured summary	2	Provide a structured summary that includes (as applicable): background, objectives, eligibility criteria, sources of evidence, charting methods, results, and conclusions that relate to the review questions and objectives.	p. 1
**INTRODUCTION**			
Rationale	3	Describe the rationale for the review in the context of what is already known. Explain why the review questions/objectives lend themselves to a scoping review approach.	p. 1 - 4
Objectives	4	Provide an explicit statement of the questions and objectives being addressed with reference to their key elements (e.g., population or participants, concepts, and context) or other relevant key elements used to conceptualize the review questions and/or objectives.	p. 3 – 4
**METHODS**			
Protocol and registration	5	Indicate whether a review protocol exists; state if and where it can be accessed (e.g., a Web address); and if available, provide registration information, including the registration number.	p. 4
Eligibility criteria	6	Specify characteristics of the sources of evidence used as eligibility criteria (e.g., years considered, language, and publication status), and provide a rationale.	p. 6 - 7
Information sources*	7	Describe all information sources in the search (e.g., databases with dates of coverage and contact with authors to identify additional sources), as well as the date the most recent search was executed.	p. 5
Search	8	Present the full electronic search strategy for at least 1 database, including any limits used, such that it could be repeated.	p. 5 - 6, and in Supplemental Materials
Selection of sources of evidence†	9	State the process for selecting sources of evidence (i.e., screening and eligibility) included in the scoping review.	p. 7
Data charting process‡	10	Describe the methods of charting data from the included sources of evidence (e.g., calibrated forms or forms that have been tested by the team before their use, and whether data charting was done independently or in duplicate) and any processes for obtaining and confirming data from investigators.	p. 8 - 9
Data items	11	List and define all variables for which data were sought and any assumptions and simplifications made.	p. 8 - 9 and in Supplemental Materials

SECTION	ITEM	PRISMA-ScR CHECKLIST ITEM	REPORTED ON PAGE #
Critical appraisal of individual sources of evidence§	12	If done, provide a rationale for conducting a critical appraisal of included sources of evidence; describe the methods used and how this information was used in any data synthesis (if appropriate).	Not applicable
Synthesis of results	13	Describe the methods of handling and summarizing the data that were charted.	p. 9
**RESULTS**			
Selection of sources of evidence	14	Give numbers of sources of evidence screened, assessed for eligibility, and included in the review, with reasons for exclusions at each stage, ideally using a flow diagram.	p. 8
Characteristics of sources of evidence	15	For each source of evidence, present characteristics for which data were charted and provide the citations.	p. 9
Critical appraisal within sources of evidence	16	If done, present data on critical appraisal of included sources of evidence (see item 12).	Not applicable
Results of individual sources of evidence	17	For each included source of evidence, present the relevant data that were charted that relate to the review questions and objectives.	p. 9 - 11
Synthesis of results	18	Summarize and/or present the charting results as they relate to the review questions and objectives.	p. 11 - 15
**DISCUSSION**			
Summary of evidence	19	Summarize the main results (including an overview of concepts, themes, and types of evidence available), link to the review questions and objectives, and consider the relevance to key groups.	p. 15 - 22
Limitations	20	Discuss the limitations of the scoping review process.	p. 20
Conclusions	21	Provide a general interpretation of the results with respect to the review questions and objectives, as well as potential implications and/or next steps.	p. 22
**FUNDING**			
Funding	22	Describe sources of funding for the included sources of evidence, as well as sources of funding for the scoping review. Describe the role of the funders of the scoping review.	p. 22

JBI = Joanna Briggs Institute; PRISMA-ScR = Preferred Reporting Items for Systematic reviews and Meta-Analyses extension for Scoping Reviews.
* Where *sources of evidence* (see second footnote) are compiled from, such as bibliographic databases, social media platforms, and Web sites.
† A more inclusive/heterogeneous term used to account for the different types of evidence or data sources (e.g., quantitative and/or qualitative research, expert opinion, and policy documents) that may be eligible in a scoping review as opposed to only studies. This is not to be confused with *information sources* (see first footnote).
‡ The frameworks by Arksey and O'Malley (6) and Levac and colleagues (7) and the JBI guidance (4, 5) refer to the process of data extraction in a scoping review as data charting.
§ The process of systematically examining research evidence to assess its validity, results, and relevance before using it to inform a decision. This term is used for items 12 and 19 instead of "risk of bias" (which is more applicable to systematic reviews of interventions) to include and acknowledge the various sources of evidence that may be used in a scoping review (e.g., quantitative and/or qualitative research, expert opinion, and policy document).

From: Tricco AC, Lillie E, Zarin W, O'Brien KK, Colquhoun H, Levac D, et al. PRISMA Extension for Scoping Reviews (PRISMAScR): Checklist and Explanation. Ann Intern Med. 2018;169:467–473. doi: 10.7326/M18-0850.

Supplementary Materials File S2) Search Query Strings

OVID Query Search String for Medline, EMBASE, PsycINFO, and Global Health Databases

Query Search String
(((sexual or gender) adj3 (orientat* or identi* or express* or divers*)) or SOGI* or SGM or GSM)<br><br>OR<br><br>(LGB* or GLBT* or LBG* or GLB* or 2SLGBT* or bisexual* or bi-sexual or bicurious* or bi-curious* or "gender* divers*" or gay? or homosexual* or lesbian* or lesbigay* or "men who have sex with men" or MSM or ((sexual* or gender* or romantic) adj3 minorit*) or non-heterosexual* or queer* or genderqueer* or "women who have sex with women" or WSW or two-spirit* or twin-spirit* or transsexual* or transgender* or transm?n or transwom?n or (trans adj2 (men or man or women or woman or boy or girl or person* or individual* or patient* or feminine or masculine)) or male-to-female or female-to-male or MTF or FTM or asexual* or aromantic* or non-binary or nonbinary or pansexual* or pan-sexual* or non-cisgender* or genderfluid* or gender-fluid* or genderless or agender or non-gendered or "third gender*" or bigender* or same-sex or "gender non conforming" or "gender non-conforming")<br><br>AND<br><br>(asylum or asylee* or claim* or "force* migra*" or "undocumented migrant*" or exile* or precari* or "precari* status")<br><br>AND<br><br>(((mental or psych* or emotional or community or spirit*) adj3 (health* or well* or illness* or condition* or support* or diagnosis or resilien*)) or depress* or anxiety or "stress disorder*" or PTSD or post-traumatic stress disorder or post traumatic stress disorder or suicid* or "suicid* ideation" or trauma or alcohol misuse or substance misuse or treatment* or therap* or psychotherap* or counsel* or "psychosocial support" or "psychosocial intervention" or "psychosocial program*")<br><br>AND<br><br>(Canad* or "British Columbia" or "Colombie Britannique" or Alberta* or Saskatchewan or Manitoba* or Ontario or Quebec or "Nouveau Brunswick" or "New Brunswick" or "Nova Scotia" or "Nouvelle Ecosse" or "Prince Edward Island" or Newfoundland or Labrador or Nunavut or NWT or "Northwest Territories" or Yukon or Nunavik or Inuvialuit)<br><br>NOT<br><br>(cancer or COPD or stroke or dialysis or diabetes or cardiovascular or parasit* or psoriasis or arthritis or vitiligo or pharma* or food or homeless* or "restless legs syndrome" or pregnancy or reproduction or tuberculosis or labor or labour or skeletal)

EBSCO Query Search String for CINAHL Plus, LGBTQ+ Source, SocIndex, ERIC, and EBSCO Open Dissertations Databases

Query Search String

(((sexual OR gender) N3 (orientat* OR identi* OR express* OR divers*)) OR SOGI* OR SGM OR GSM)

OR

(LGB* OR GLBT* OR LBG* OR GLB* OR 2SLGBT* OR bisexual* OR bi-sexual OR bicurious* OR bi-curious* OR "gender* divers*" OR gay# OR homosexual* OR lesbian* OR lesbigay* OR "men who have sex with men" OR MSM OR ((sexual* OR gender* OR romantic) N3 minorit*) OR non-heterosexual* OR queer* OR genderqueer* OR "women who have sex with women" OR WSW OR two-spirit* OR twin-spirit* OR transsexual* OR transgender* OR transm#n OR transwom#n OR (trans N2 (men OR man OR women OR woman OR boy OR girl OR person* OR individual* OR patient* OR feminine OR masculine)) OR male-to-female OR female-to-male OR MTF OR FTM OR asexual* OR aromantic* OR non-binary OR nonbinary OR pansexual* OR pan-sexual* OR non-cisgender* OR genderfluid* OR gender-fluid* OR genderless OR agender OR non-gendered OR "third gender*" OR bigender* OR same-sex OR "gender non conforming" OR "gender non-conforming")

AND

(asylum OR asylee* OR claim* OR "force* migra*" OR "undocumented migrant*" OR exile* OR precari* OR "precari* status")

AND

(((mental OR psych* OR emotional OR community OR spirit*) N3 (health* OR well* OR illness* OR condition* OR support* OR diagnosis OR resilien*)) OR depress* OR anxiety OR "stress disorder*" OR PTSD OR "post-traumatic stress disorder" OR "post traumatic stress disorder" OR suicid* OR "suicid* ideation" OR trauma OR "alcohol misuse" OR "substance misuse" OR treatment* OR therap* OR psychotherap* OR counsel* OR "psychosocial support" OR "psychosocial intervention" OR "psychosocial program*")

AND

(Canad* OR "British Columbia" OR "Colombie Britannique" OR Alberta* OR Saskatchewan OR Manitoba* OR Ontario OR Quebec OR "Nouveau Brunswick" OR "New Brunswick" OR "Nova Scotia" OR "Nouvelle Ecosse" OR "Prince Edward Island" OR Newfoundland OR Labrador OR Nunavut OR NWT OR "Northwest Territories" OR Yukon OR Nunavik OR Inuvialuit)

NOT

(cancer OR COPD OR stroke OR dialysis OR diabetes OR cardiovascular OR parasit* OR psoriasis OR arthritis OR vitiligo OR pharma* OR food OR homeless* OR "restless legs syndrome" OR pregnancy OR reproduction OR tuberculosis OR labor OR labour OR skeletal)

Elsevier Query String for Scopus Database

Query Search String

(((sexual OR gender) W/3 (orientat* OR identi* OR express* OR divers*)) OR SOGI* OR SGM OR GSM)

OR

(LGB* OR GLBT* OR LBG* OR GLB* OR 2SLGBT* OR bisexual* OR bi-sexual OR bicurious* OR bi-curious* OR "gender* divers*" OR gay* OR homosexual* OR lesbian* OR lesbigay* OR "men who have sex with men" OR MSM OR ((sexual* OR gender* OR romantic) W/3 minorit*) OR non-heterosexual* OR queer* OR genderqueer* OR "women who have sex with women" OR WSW OR two-spirit* OR twin-spirit* OR transsexual* OR transgender* OR transm*n OR transwom*n OR (trans W/2 (men OR man OR women OR woman OR boy OR girl OR person* OR individual* OR patient* OR feminine OR masculine)) OR male-to-female OR female-to-male OR MTF OR FTM OR asexual* OR aromantic* OR non-binary OR nonbinary OR pansexual* OR pan-sexual* OR non-cisgender* OR genderfluid* OR gender-fluid* OR genderless OR agender OR non-gendered OR "third gender*" OR bigender* OR same-sex OR "gender non conforming" OR "gender non-conforming")

AND

(asylum OR asylee* OR claim* OR "force* migra*" OR "undocumented migrant*" OR exile* OR precari* OR "precari* status")

AND

(((mental OR psych* OR emotional OR community OR spirit*) W/3 (health* OR well* OR illness* OR condition* OR support* OR diagnosis OR resilien*)) OR depress* OR anxiety OR "stress disorder*" OR PTSD OR "post-traumatic stress disorder" OR "post traumatic stress disorder" OR suicid* OR "suicid* ideation" OR trauma OR "alcohol misuse" OR "substance misuse" OR treatment* OR therap* OR psychotherap* OR counsel* OR "psychosocial support" OR "psychosocial intervention" OR "psychosocial program*")

AND

(Canad* OR "British Columbia" OR "Colombie Britannique" OR Alberta* OR Saskatchewan OR Manitoba* OR Ontario OR Quebec OR "Nouveau Brunswick" OR "New Brunswick" OR "Nova Scotia" OR "Nouvelle Ecosse" OR "Prince Edward Island" OR Newfoundland OR Labrador OR Nunavut OR NWT OR "Northwest Territories" OR Yukon OR Nunavik OR Inuvialuit)

NOT

(cancer OR COPD OR stroke OR dialysis OR diabetes OR cardiovascular OR parasit* OR psoriasis OR arthritis OR vitiligo OR pharma* OR food OR homeless* OR "restless legs syndrome" OR pregnancy OR reproduction OR tuberculosis OR labor OR labour OR skeletal)

Clarivate Query String for Web of Science and Proquest Dissertation and Theses Citation Index Databases

Query Search String
(((sexual OR gender) NEAR/3 (orientat* OR identi* OR express* OR divers*)) OR SOGI* OR SGM OR GSM)<br><br>OR<br><br>(LGB* OR GLBT* OR LBG* OR GLB* OR 2SLGBT* OR bisexual* OR bi-sexual OR bicurious* OR bi-curious* OR "gender* divers*" OR gay$ OR homosexual* OR lesbian* OR lesbigay* OR "men who have sex with men" OR MSM OR ((sexual* OR gender* OR romantic) NEAR/3 minorit*) OR non-heterosexual* OR queer* OR genderqueer* OR "women who have sex with women" OR WSW OR two-spirit* OR twin-spirit* OR transsexual* OR transgender* OR transm$n OR transwom$n OR (trans NEAR/2 (men OR man OR women OR woman OR boy OR girl OR person* OR individual* OR patient* OR feminine OR masculine)) OR male-to-female OR female-to-male OR MTF OR FTM OR asexual* OR aromantic* OR non-binary OR nonbinary OR pansexual* OR pan-sexual* OR non-cisgender* OR genderfluid* OR gender-fluid* OR genderless OR agender OR non-gendered OR "third gender*" OR bigender* OR same-sex OR "gender non conforming" OR "gender non-conforming")<br><br>AND<br><br>(asylum OR asylee* OR claim* OR "force* migra*" OR "undocumented migrant*" OR exile* OR precari* OR "precari* status")<br><br>AND<br><br>(((mental OR psych* OR emotional OR community OR spirit*) NEAR/3 (health* OR well* OR illness* OR condition* OR support* OR diagnosis OR resilien*)) OR depress* OR anxiety OR "stress disorder*" OR PTSD OR "post-traumatic stress disorder" OR "post traumatic stress disorder" OR suicid* OR "suicid* ideation" OR trauma OR "alcohol misuse" OR "substance misuse" OR treatment* OR therap* OR psychotherap* OR counsel* OR "psychosocial support" OR "psychosocial intervention" OR "psychosocial program*")<br><br>AND<br><br>(Canad* OR "British Columbia" OR "Colombie Britannique" OR Alberta* OR Saskatchewan OR Manitoba* OR Ontario OR Quebec OR "Nouveau Brunswick" OR "New Brunswick" OR "Nova Scotia" OR "Nouvelle Ecosse" OR "Prince Edward Island" OR Newfoundland OR Labrador OR Nunavut OR NWT OR "Northwest Territories" OR Yukon OR Nunavik OR Inuvialuit)<br><br>NOT<br><br>(cancer OR COPD OR stroke OR dialysis OR diabetes OR cardiovascular OR parasit* OR psoriasis OR arthritis OR vitiligo OR pharma* OR food OR homeless* OR "restless legs syndrome" OR pregnancy OR reproduction OR tuberculosis OR labor OR labour OR skeletal)

Proquest Advanced Query String for Canadian Research Index and Sociological Abstracts

Query Search String
(((sexual OR gender) NEAR/3 (orientat* OR identi* OR express* OR divers*)) OR SOGI* OR SGM OR GSM)<br><br>OR<br><br>(LGB* OR GLBT* OR LBG* OR GLB* OR 2SLGBT* OR bisexual* OR bi-sexual OR bicurious* OR bi-curious* OR "gender* divers*" OR gay* OR homosexual* OR lesbian* OR lesbigay* OR "men who have sex with men" OR MSM OR ((sexual* OR gender* OR romantic) NEAR/3 minorit*) OR non-heterosexual* OR queer* OR genderqueer* OR "women who have sex with women" OR WSW OR two-spirit* OR twin-spirit* OR transsexual* OR transgender* OR transman OR transmen OR transwoman OR transwomen OR (trans NEAR/2 (men OR man OR women OR woman OR boy OR girl OR person* OR individual* OR patient* OR feminine OR masculine)) OR male-to-female OR female-to-male OR MTF OR FTM OR asexual* OR aromantic* OR non-binary OR nonbinary OR pansexual* OR pan-sexual* OR non-cisgender* OR genderfluid* OR gender-fluid* OR genderless OR agender OR non-gendered OR "third gender*" OR bigender* OR same-sex OR "gender non conforming" OR "gender non-conforming")<br><br>AND<br><br>(asylum OR asylee* OR claim* OR "force* migra*" OR "undocumented migrant*" OR exile* OR precari* OR "precari* status")<br><br>AND<br><br>(((mental OR psych* OR emotional OR community OR spirit*) NEAR/3 (health* OR well* OR illness* OR condition* OR support* OR diagnosis OR resilien*)) OR depress* OR anxiety OR "stress disorder*" OR PTSD OR "post-traumatic stress disorder" OR "post traumatic stress disorder" OR suicid* OR "suicid* ideation" OR trauma OR "alcohol misuse" OR "substance misuse" OR treatment* OR therap* OR psychotherap* OR counsel* OR "psychosocial support" OR "psychosocial intervention" OR "psychosocial program*")<br><br>AND<br><br>(Canad* OR "British Columbia" OR "Colombie Britannique" OR Alberta* OR Saskatchewan OR Manitoba* OR Ontario OR Quebec OR "Nouveau Brunswick" OR "New Brunswick" OR "Nova Scotia" OR "Nouvelle Ecosse" OR "Prince Edward Island" OR Newfoundland OR Labrador OR Nunavut OR NWT OR "Northwest Territories" OR Yukon OR Nunavik OR Inuvialuit)<br><br>NOT<br><br>(cancer OR COPD OR stroke OR dialysis OR diabetes OR cardiovascular OR parasit* OR psoriasis OR arthritis OR vitiligo OR pharma* OR food OR homeless* OR "restless legs syndrome" OR pregnancy OR reproduction OR tuberculosis OR labor OR labour OR skeletal)

Supplementary Materials File S3) Grey Literature Search Plan Record

The following tables list the grey literature search process for targeted websites and browsing (Table S3A), specific grey literature databases (Table S3B), and Google searches (Table S3C). For Table S3A, aside from UNHCR Canada and Egale, the organizations listed were based on the directory of member organizations for the Canadian Council for Refugees.

This grey literature search plan template has been slightly modified to meet the needs of this scoping review project and was originally created by Jackie Stapleton (2018) from the University of Waterloo.

Table S3A

Targeted Website Browsing/Search Records

Organization name	URL	Date	Search Terms Used	# items ID'd for full screening	General Comments
UNHCR Canada	https://www.unhcr.ca/	March 8, 2026	LGBT asylum (49 results)	0	Articles did not have any that addressed LGBT asylum seeker mental health
Egale	https://egale.ca/	March 8, 2026	Asylum (10 search results)<br>*(Given this was an LGBTQ-focsed organization, chose to focus on the RAS aspect of search terms)*	1	
Action Coalition on Human Trafficking (ACT) Alberta	https://www.actalberta.org/	March 8, 2026	No search function provided. Looked around the webpage.	N/A	N/A
Alberta Association of Immigrant Serving Agencies	https://aaisa.ca/	March 8, 2026	LGBT asylum (2 results)	0	None addressed LGBT asylum seeker mental health

Organization name	URL	Date	Search Terms Used	# items ID'd for full screening	General Comments
Calgary Catholic Immigration Society	https://ccisab.ca/	March 8, 2026	LGBT asylum (0 results)	0	N/A
Canadian Immigrant Women's Association	https://ciwa-online.com/	March 8, 2026	LGBT asylum (0 results)	0	N/A
Canadian Baptists of Western Canada	https://cbwc.ca/	March 8, 2026	LGBT asylum (0 results)	0	N/A
Catholic Social Services	https://www.cssalberta.ca/	March 8, 2026	No search function provided. Looked around the webpage.	N/A	Could not find items related to LGBTQ+ asylum seeker mental health
Edmonton Newcomer Centre	https://newcomercentre.com/	March 8, 2026	LGBT asylum (10 results)	0	None addressed LGBT asylum seeker mental health
End of the Rainbow Foundation	https://endoftherainbow.ca/	March 8, 2026	No search function provided. Looked around the webpage.	N/A	Could not find items related to LGBTQ+ asylum seeker mental health
Francophonie Albertaine Plurielle (FRAP)	https://frap.ca/	March 8, 2026	N/A	N/A	Website was in French
Immigrant Services Calgary	https://www.immigrantservicescalgary.ca/	March 8, 2026	LGBT asylum (2 results)	0	None addressed LGBT asylum seeker mental health
Islamic Family and Social Services Association (IFSSA)	https://www.islamicfamily.ca/	March 8, 2026	LGBT asylum (0 results)	0	N/A

Organization name	URL	Date	Search Terms Used	# items ID'd for full screening	General Comments
Manmeet Singh Bhullar Foundation	https://www.themsbf.ca/	March 8, 2026	No search function provided. Looked around the webpage.	N/A	Could not find items related to LGBTQ+ asylum seeker mental health
Migrante Canada	https://www.migrante.ca/	March 8, 2026	No search function provided. Looked around the webpage.	N/A	Could not find items related to LGBTQ+ asylum seeker mental health
Roman Catholic Diocese of Calgary	https://www.catholicyyc.ca/	March 8, 2026	No search function provided. Looked around the webpage.	N/A	Could not find items related to LGBTQ+ asylum seeker mental health
Affiliation of Multicultural Societies & Service Agencies of British Columbia (AMSSA)	https://www.amssa.org/	March 8, 2026	LGBT asylum (0 results)	0	N/A
Anglican Synod of the Diocese of BC - Refugee Program	https://bc.anglican.ca/	March 8, 2026	LGBT asylum (0 results)	0	N/A
Battered Women's Support Services (BWSS)	https://www.bwss.org/	March 8, 2026	LGBT asylum (1 result)	0	None addressed LGBT asylum seeker mental health
Canadian Lutheran World Relief Refugee Program	https://www.clwr.org/	March 8, 2026	LGBT asylum (0 results)	0	N/A
Chimo Community Services	https://chimoservices.com/	March 8, 2026	LGBT asylum (0 results)	0	N/A

Organization name	URL	Date	Search Terms Used	# items ID'd for full screening	General Comments
Cowichan Intercultural Society (CIS)	https://www.cowichanintercultural.org/	March 8, 2026	No search function provided. Looked around the webpage.	N/A	Could not find items related to LGBTQ+ asylum seeker mental health
Diocese of New Westminster (Refugee Unit)	https://www.vancouver.anglican.ca/	March 8, 2026	LGBT asylum (1 result)	0	None addressed LGBT asylum seeker mental health
DIVERSECity Community Resources Society	https://www.dcrs.ca/	March 8, 2026	LGBT asylum (44 results)	0	None addressed LGBT asylum seeker mental health
East Kootenay Friends of Refugees Society	Link did not work	March 8, 2026	N/A	N/A	N/A
H.E.R.S FOUNDATION	http://www.hersvictoria.com/	March 8, 2026	No search function provided. Looked around the webpage.	N/A	N/A
Immigrant Services Society of B.C.	https://issbc.org/	March 8, 2026	LGBT asylum (0 results)	0	N/A
Inasmuch Community Society	https://www.inasmuch.ca/	March 8, 2026	No search function provided. Looked around the webpage.	N/A	Could not find items related to LGBTQ+ asylum seeker mental health
Inter-Cultural Association of Greater Victoria	https://www.icavictoria.org/	March 8, 2026	LGBT asylum (0 results)	0	N/A

Organization name	URL	Date	Search Terms Used	# items ID'd for full screening	General Comments
Journey Home Community Association	https://journeyhomecommunity.ca/	March 8, 2026	No search function provided. Looked around the webpage.	N/A	Could not find items related to LGBTQ+ asylum seeker mental health
Kinbrace Community Society	https://kinbrace.ca/	March 8, 2026	No search function provided. Looked around the webpage.	N/A	Could not find items related to LGBTQ+ asylum seeker mental health
MOSAIC	https://mosaicbc.org/	March 8, 2026	LGBT asylum (3 results)	1	Has program description for supports for LGBTQ+ asylum seekers
Mothers Matter Centre (MMC)	Website no longer exists	March 8, 2026	N/A	N/A	N/A
Options Community Services Society - Immigrant Services Division	https://www.options.bc.ca/	March 8, 2026	LGBT asylum (0 results)	N/A	N/A
Pacific Immigrant Resources Society	https://pirs.bc.ca/	March 8, 2026	LGBT asylum (0 results)	N/A	N/A
Rainbow Refugee Society	https://rainbowrefugee.ca/	March 8, 2026	No search function provided	0	Explored website, does have settlement supports for LGBTQ+ refugee and asylum seekers, but nothing noted about mental health specifically for scoping review
RED Legal	Website no longer exists	March 8, 2026	N/A	N/A	N/A

Organization name	URL	Date	Search Terms Used	# items ID'd for full screening	General Comments
S.U.C.C.E.S.S.	https://successbc.ca/	March 8, 2026	LGBT asylum (0 results)	N/A	N/A
The Global Alliance Against Traffic in Women Canada (GAATW)	https://gaatw.ca/	March 8, 2026	No search function provided	N/A	Could not find items related to LGBTQ+ asylum seeker mental health
Vancouver Association for the Survivors of Torture (VAST)	https://www.vastbc.ca/	March 8, 2026	No search function provided	N/A	Had supports for refugee and asylum seekers, but none for LGBTQ+
Vancouver Island Counselling Centre for Immigrants and Refugees (VICCIR)	https://www.viccir.org/	March 8, 2026	No search function provided	N/A	Could not find items related to LGBTQ+ asylum seeker mental health
Victoria Immigrant and Refugee Centre Society (VIRCS)	https://www.vircs.bc.ca/	March 8, 2026	LGBT asylum (0 results)	0	N/A
West Kootenay Friends of Refugees	Website no longer exists	March 8, 2026	N/A	N/A	N/A
YWCA Metro Vancouver - Safe Heaven Project	https://ywcabc.org/	March 8, 2026	LGBT asylum (0 results)	0	N/A
Yukon Cares	https://yukoncares.ca/	March 8, 2026	No search function provided	N/A	Could not find items related to LGBTQ+ asylum seeker mental health

Organization name	URL	Date	Search Terms Used	# items ID'd for full screening	General Comments
Anglican Diocese of Saskatoon Diocesan Refugee Committee	https://www.anglicandiocesesaskatoon.com/refugee	March 8, 2026	No search function provided	N/A	Could not find items related to LGBTQ+ asylum seeker mental health
Regina Open Door Society Inc.	https://rods.sk.ca/	March 8, 2026	LGBT asylum (0 results)	0	N/A
Roman Catholic Diocese of Saskatoon (Episcopal Corp. of Saskatoon) - Migration Office	https://saskatoonrcdiocese.com/	March 8, 2026	LGBT asylum (2 results)	0	None addressed LGBT asylum seeker mental health
SAISIA: Saskatchewan Association of Immigrant Settlement and Integration Agencies	https://saisia.ca/	March 8, 2026	LGBT asylum (0 results)	0	N/A
Saskatoon Open Door Society Inc.	https://sods.sk.ca/	March 8, 2026	LGBT asylum (0 results)	0	N/A
Saskatoon Refugee Coalition	https://www.facebook.com/SaskatoonRefugeeCoalition/	March 8, 2026	No search function provided	N/A	Could not find items related to LGBTQ+ asylum seeker mental health
Accueil Liaison Pour Arrivants (ALPA)	https://www.alpaong.com/	March 8, 2026	No search function provided	N/A	Could not find items related to LGBTQ+ asylum seeker mental health

Organization name	URL	Date	Search Terms Used	# items ID'd for full screening	General Comments
Accueil pour immigrants et réfugiés du Sud-ouest de Montréal / Centre prisme	https://www.airsomprisme.org/	March 8, 2026	No search function provided	N/A	Could not find items related to LGBTQ+ asylum seeker mental health
Accueil-Parrainage Outaouais	https://apo-qc.org/	March 8, 2026	N/A	N/A	Website in French
Action des chretiens-nes pour l'abolition de la torture ACAT - Canada	https://acatcanada.ca/	March 8, 2026	N/A	N/A	Website in French
Action Réfugiés Montréal	https://actionr.org/	March 8, 2026	LGBT asylum (3 results)	0	None addressed LGBT asylum seeker mental health
AGIR: Action Lesbienne, Gai, Bisexuelle, Trans et Queer (LGBTQ) avec les immigrants et réfugiés	https://agirmontreal.org/en/education/	March 8, 2026	Looked at their resources sections	1	Had a resource addressing LGBT refugee and asylum seeker experiences for further screening
Alternatives	https://www.alternatives.ca/	March 8, 2026	N/A	N/A	Website in French
Amnistie internationale Canada - Section francophone	https://amnistie.ca/	March 8, 2026	N/A	N/A	Website in French

Organization name	URL	Date	Search Terms Used	# items ID'd for full screening	General Comments
Archevêché de Montréal Office des Communautés Culturelles	https://www.diocesemontreal.org/fr	March 8, 2026	N/A	N/A	Website in French
Association des lesbiennes et des gais sur Internet (ALGI)	https://www.algi.qc.ca/	March 8, 2026	N/A	N/A	Website in French
Association pour les Droits de Travailleuses.rs de Maison et de Ferme (DTMF)	https://dtmf-rhfw.org/	March 8, 2026	N/A	N/A	Website in French
Association québécoise des avocats et avocates en droit de l'immigration (AQAADI)	https://dtmf-rhfw.org/	March 8, 2026	N/A	N/A	Website link no longer works
Carrefour d'action interculturelle	http://www.soutenir.org/	March 8, 2026	N/A	N/A	Website link no longer works
Carrefour d'aide aux nouveaux arrivants (CANA)	https://canamtl.com/	March 8, 2026	N/A	N/A	Website in French
Carrefour de ressources en interculturel (CRIC)	https://cricentresud.org/	March 8, 2026	N/A	N/A	Website in French

Organization name	URL	Date	Search Terms Used	# items ID'd for full screening	General Comments
Centre communautaire des femmes Sud-asiatique	https://www.sawcc-ccfsa.ca/EN/	March 8, 2026	No search function provided. Looked around the webpage.	N/A	Could not find items related to LGBTQ+ asylum seeker mental health
Centre communautaire LGBTQ+ de Montréal	https://ccglm.org/en/	March 8, 2026	No search function provided. Looked around the webpage.	N/A	Could not find items related to LGBTQ+ asylum seeker mental health
Centre d'hébergement La Traverse	Could not find website link	March 8, 2026	N/A	N/A	N/A
Centre d'orientation paralégale et sociale pour immigrants (COPSI)	https://www.copsi.ca/en	March 8, 2026	No search function provided. Looked around the webpage.	N/A	Could not find items related to LGBTQ+ asylum seeker mental health
Centre Justice et Foi, secteur "Vivre Ensemble"	https://cjf.qc.ca/vivre-ensemble/	March 8, 2026	No search function provided. Looked around the webpage.	N/A	Could not find items related to LGBTQ+ asylum seeker mental health
Centre multiethnique de Québec	https://www.centremultiethnique.com/	March 8, 2026	N/A	N/A	Website in French
Centre social d'aide aux immigrants (CSAI)	https://centrecsai.org/en/centre-csai-en/	March 8, 2026	No search function provided. Looked around the webpage.	N/A	Could not find items related to LGBTQ+ asylum seeker mental health

Organization name	URL	Date	Search Terms Used	# items ID'd for full screening	General Comments
Clinique pour la justice migrante	https://www.cliniquejusticemigrante.org/en	March 8, 2026	No search function provided. Looked around the webpage.	N/A	Could not find items related to LGBTQ+ asylum seeker mental health
Coalition québécoise contre la traite des personnes	https://www.cqctp.org/	March 8, 2026	N/A	N/A	Website in French
Comité d'accueil international des Bois-Francs	https://www.caibf.ca/	March 8, 2026	N/A	N/A	Website in French
Comité d'action contre la traite humaine interne et internationale (CATHII)	https://www.cathii.org/	March 8, 2026	N/A	N/A	Website in French
Comité d'aide aux réfugiés	N/A	March 8, 2026	N/A	N/A	Could not find link
Foyer du Monde	https://foyerdumonde.ca/	March 8, 2026	N/A	N/A	Website in French
Immigrant Workers Centre	https://iwc-cti.ca/	March 8, 2026	LGBT asylum (1 result)	0	None addressed LGBT asylum seeker mental health
Jesuit Refugee Service	https://canada.jrs.net/en/home/	March 8, 2026	LGBT asylum (0 results)	0	N/A

Organization name	URL	Date	Search Terms Used	# items ID'd for full screening	General Comments
La Société John Howard du Québec	https://john-howard.qc.ca/fr/	March 8, 2026	N/A	N/A	Website in French
Le COFFRET	https://www.lecoffret.org/	March 8, 2026	N/A	N/A	Website in French
Le Phare des AffranchiEs	https://en.affranchies.ca/	March 8, 2026	No search function provided. Looked around the webpage.	N/A	Could not find items related to LGBTQ+ asylum seeker mental health
Le Pont – APPI (Accueillir, Protéger, Promouvoir, Intégrer)	N/A	March 8, 2026	N/A	N/A	Website link no longer active
Montreal City Mission	https://www.montrealcitymission.org/	March 8, 2026	LGBT asylum (6 results)	0	None addressed LGBT asylum seeker mental health
Motivaction Jeunesse	https://motivactionjeunesse.com/	March 8, 2026	N/A	N/A	Website in French
Mouvement contre le viol et l'inceste (MCVI)	https://www.mcvicontreleviol.org/	March 8, 2026	N/A	N/A	Website in French
PINAY	http://www.pinayquebec.blogspot.com/	March 8, 2026	N/A	N/A	Website could not be accessed

Organization name	URL	Date	Search Terms Used	# items ID'd for full screening	General Comments
Project Genesis	https://genese.qc.ca/	March 8, 2026	No search function provided. Looked around the webpage.	N/A	Could not find items related to LGBTQ+ asylum seeker mental health
Regroupement des organismes en hébergement pour personnes migrantes (ROHMI)	https://rohmi.ca/	March 8, 2026	N/A	N/A	Website in French
Réseau d'aide aux travailleuses et travailleurs migrants agricoles du Québec (RATTMAQ)	https://rattmaq.org/	March 8, 2026	N/A	N/A	Website in French
Réseau d'intervention auprès des pers. ayant subi la violence organisée (RIVO)	https://rivo-resilience.org/	March 8, 2026	N/A	N/A	Website in French
Say Ça! Circle of support for refugee and newcomer students	https://www.saycamontreal.ca/	March 8, 2026	No search function provided. Looked around the webpage.	N/A	Could not find items related to LGBTQ+ asylum seeker mental health
Service d'aide aux néo-canadiens Sherbrooke (SANC)	https://www.sanc-sherbrooke.ca/	March 8, 2026	N/A	N/A	Website in French

Organization name	URL	Date	Search Terms Used	# items ID'd for full screening	General Comments
Service d'éducation et d'intégration interculturelles de Montréal (SEIIM)	https://seiim.org/	March 8, 2026	N/A	N/A	Website in French
SINGA Québec : innovation social et enrichissement culturel avec les réfugiés	https://www.singa.quebec/	March 8, 2026	N/A	N/A	Website in French
Table de Concertation des organismes au service des personnes Réfugiées et Immigrantes (TCRI)	https://tcri.qc.ca/	March 8, 2026	N/A	N/A	Website in French
The Good Samaritan Relief Fund	N/A	March 8, 2026	N/A	N/A	Could not find website link
The Refugee Centre	https://www.therefugeecentre.org/home	March 8, 2026	No search function provided. Looked around the webpage.	N/A	Could not find items related to LGBTQ+ asylum seeker mental health
The Welcome Collective	https://www.welcomecollective.org/	March 8, 2026	No search function provided. Looked around the webpage.	N/A	Could not find items related to LGBTQ+ asylum seeker mental health. They specifically serve refugee claimants.
Cooper Institute	https://www.cooperinstitute.ca/	March 8, 2026	No search function provided. Looked around the webpage.	N/A	Could not find items related to LGBTQ+ asylum seeker mental health

Organization name	URL	Date	Search Terms Used	# items ID'd for full screening	General Comments
Immigrant & Refugee Services Association PEI - IRSA PEI	https://www.irsapei.ca/en/home	March 8, 2026	LGBT asylum (2 results)	0	None addressed LGBT asylum seeker mental health
A & O: Support Services for Older Adults Inc.	https://www.aosupportservices.ca/	March 8, 2026	No search function provided. Looked around the webpage.	N/A	Could not find items related to LGBTQ+ asylum seeker mental health
Accueil francophone du Manitoba	http://www.accueilfrancophonemb.com/	March 8, 2026	N/A	N/A	Website in French
Aurora Family Therapy Centre Inc.	https://resiliacentre.ca/	March 8, 2026	No search function provided. Looked around the webpage.	N/A	Could not find items related to LGBTQ+ asylum seeker mental health
Healthy Muslim Families (HMF)	https://healthymuslimfamilies.ca/	March 8, 2026	No search function provided. Looked around the webpage.	N/A	Could not find items related to LGBTQ+ asylum seeker mental health
Hospitality House Refugee Ministry Inc.	https://hhrmwpg.org/	March 8, 2026	No search function provided. Looked around the webpage.	N/A	Could not find items related to LGBTQ+ asylum seeker mental health
Immigrant & Refugee Community Organization of Manitoba Inc. (IRCOM INC.)	https://ircom.ca/	March 8, 2026	No search function provided. Looked around the webpage.	N/A	Could not find items related to LGBTQ+ asylum seeker mental health
Jewish Child & Family Service - Settlement Services	https://jcfswinnipeg.org/	March 8, 2026	No search function provided. Looked around the webpage.	N/A	Could not find items related to LGBTQ+ asylum seeker mental health

Organization name	URL	Date	Search Terms Used	# items ID'd for full screening	General Comments
Manitoba Association of Newcomer Serving Organizations (MANSO)	https://mansomanitoba.ca/	March 8, 2026	No search function provided. Looked around the webpage.	N/A	Could not find items related to LGBTQ+ asylum seeker mental health. Did have a section for refugee claimants resources.
Manitoba Association of Women's Shelters Inc. (MAWS)	https://maws.mb.ca/	March 8, 2026	No search function provided. Looked around the webpage.	N/A	Could not find items related to LGBTQ+ asylum seeker mental health
Manitoba Interfaith Immigration Council Inc.	https://www.miic.ca/	March 8, 2026	No search function provided. Looked around the webpage.	N/A	Could not find items related to LGBTQ+ asylum seeker mental health. Did have a section for refugee claimants resources.
Mennonite Central Committee Canada	https://mcc.org/	March 8, 2026	LGBT asylum (30 results)	0	None addressed LGBT asylum seeker mental health
Mosaic Newcomer Family Resource Network	https://www.mosaicnet.ca/	March 8, 2026	LGBT asylum (0 results)	0	N/A
Mount Carmel Clinic - Multicultural Wellness Program	https://www.mountcarmel.ca/	March 8, 2026	No search function provided. Looked around the webpage.	N/A	Could not find items related to LGBTQ+ asylum seeker mental health
New Journey Housing	https://newjourneyhousing.com/	March 8, 2026	No search function provided. Looked around the webpage.	N/A	Could not find items related to LGBTQ+ asylum seeker mental health
Newcomers Employment & Education Development Services (NEEDS) Inc.	https://www.needsinc.ca/	March 8, 2026	No search function provided. Looked around the webpage.	N/A	Could not find items related to LGBTQ+ asylum seeker mental health

Organization name	URL	Date	Search Terms Used	# items ID'd for full screening	General Comments
North End Sponsorship Team (NEST)	N/A	March 8, 2026	N/A	N/A	Could not find the website link
Regional Connections Immigrant Services	https://regionalconnections.ca/	March 8, 2026	No search function provided. Looked around the webpage.	N/A	Could not find items related to LGBTQ+ asylum seeker mental health
Somali Help-Age Association	N/A	March 8, 2026	N/A	N/A	Could not find the website link
Asian Heritage Society of NB	https://www.ahsnb.org/	March 8, 2026	LGBT asylum (0 results)	0	N/A
Immigrant and Refugee Support Centre	Website link not found	March 8, 2026	N/A	N/A	N/A
Madhu Verma Migrant Justice Centre (Madhu Centre)	https://www.madhucentre.ca/	March 8, 2026	No search function provided. Looked around the webpage.	N/A	Could not find items related to LGBTQ+ asylum seeker mental health
Multicultural Association of Fredericton Inc.	https://mcaf.nb.ca/en/	March 8, 2026	No search function provided. Looked around the webpage.	N/A	Had an asylum seeker section, but did not have anything related to LGBTQ+ asylum seeker mental health
New Brunswick Multicultural Council	https://www.nbmc-cmnb.ca/	March 8, 2026	LGBT asylum (1 result)	0	None addressed LGBT asylum seeker mental health
New Brunswick Refugee Clinic	https://nbrc-crnb.ca/	March 8, 2026	No search function provided. Looked around the webpage.	N/A	Could not find items related to LGBTQ+ asylum seeker mental health

Organization name	URL	Date	Search Terms Used	# items ID'd for full screening	General Comments
Newcomer Connections, YMCA Southwestern New Brunswick	https://swnb.ymca.ca/	March 8, 2026	No search function provided. Looked around the webpage.	N/A	Could not find items related to LGBTQ+ asylum seeker mental health
Association for New Canadians (ANC)	https://ancnl.ca/	March 8, 2026	LGBT asylum (1 result)	0	None addressed LGBT asylum seeker mental health
Atlantic Region Association of Immigrant Serving Agencies (ARAISA)	https://araisa.ca/	March 8, 2026	LGBT asylum (5 results)	1	Had information on supporting LGBTQ+ newcomers and mental health for further screening
Halifax Refugee Clinic	https://halifaxrefugeeclinic.org/	March 8, 2026	No search function provided. Looked around the webpage.	N/A	Could not find items related to LGBTQ+ asylum seeker mental health
Immigrant Services Association of Nova Scotia (ISANS)	https://isans.ca/	March 8, 2026	LGBT asylum (1 result)	1	Included some mental health for LGBTQ+ newcomers for further screening
Immigration Francophone de Nouvelle-Écosse (Fédération acadienne de la Nouvelle-Écosse)	https://ifne.ca/	March 8, 2026	N/A	N/A	Website in French
Rainbow Refugee Association of Nova Scotia	https://www.rainbowrefugeens.com/	March 8, 2026	Searched through organizational resources	0	None addressed LGBT asylum seeker mental health interventions specifically (only general resources available)

Organization name	URL	Date	Search Terms Used	# items ID'd for full screening	General Comments
Access Alliance Multicultural Health and Community Services	http://www.accessalliance.ca/	March 8, 2026	LGBT asylum (6 results)	1	Included reference to LGBTQ+ newcomer mental health for further screening
Adam House	https://www.adamhouse.org/	March 8, 2026	No search function provided. Looked around the webpage.	N/A	Could not find items related to LGBTQ+ asylum seeker mental health
Afghan Women's Organization Refugee and Immigrant Services	https://afghanwomen.org/	March 8, 2026	No search function provided. Looked around the webpage.	N/A	Could not find items related to LGBTQ+ asylum seeker mental health
Afro Canadian Development Inc. (ACD)	https://afrocanadiandevelopment.org/	March 8, 2026	LGBT asylum (0 results)	0	N/A
Alliance des Burundais du Canada	https://abuca.org/en/about-us/	March 8, 2026	No search function provided. Looked around the webpage.	N/A	Could not find items related to LGBTQ+ asylum seeker mental health
Alongside Hope (formerly the Primate's World Relief and Development Fund)	https://alongsidehope.org/	March 8, 2026	LGBT asylum (23 results)	0	None addressed LGBT asylum seeker mental health
Amnesty International	https://amnesty.ca/	March 8, 2026	LGBT asylum (54 results)	0	None addressed LGBT asylum seeker mental health
Angela Rose House	Website link not provided	March 8, 2026	N/A	N/A	N/A

Organization name	URL	Date	Search Terms Used	# items ID'd for full screening	General Comments
Anglican United Refugee Alliance (AURA)	https://aurafforrefugees.org/	March 8, 2026	No search function provided. Looked around the webpage.	N/A	Could not find items related to LGBTQ+ asylum seeker mental health
Arab Community Centre of Toronto	https://acctonline.ca/	March 8, 2026	No search function provided. Looked around the webpage.	N/A	Could not find items related to LGBTQ+ asylum seeker mental health
Barbra Schlifer Commemorative Clinic	https://www.schliferclinic.com/resources-toolkits/	March 8, 2026	No search function provided. Looked around the webpage.	N/A	Could not find items related to LGBTQ+ asylum seeker mental health
Black Africanness Regulated Canadian Immigration Consultants (BA-RCIC)	https://ba-rcic.com/	March 8, 2026	No search function provided. Looked around the webpage.	N/A	Could not find items related to LGBTQ+ asylum seeker mental health
Camille Kamanzi Foundation	https://www.ckfoundation.ca/	March 8, 2026	N/A	N/A	Website link not working, no others found
Canada Excellence Research Chair in Migration and Integration (CERC)	https://www.torontomu.ca/cerc-migration/	March 8, 2026	Looked in webpage	0	Could not find items related to LGBTQ+ asylum seeker mental health
Canadian Association of Refugee Lawyers (CARL)	https://carl-acaadr.ca/	March 8, 2026	No search function provided. Looked around the webpage.	N/A	Could not find items related to LGBTQ+ asylum seeker mental health

Organization name	URL	Date	Search Terms Used	# items ID'd for full screening	General Comments
Canadian Baptists of Ontario & Quebec	https://baptist.ca/	March 8, 2026	Search function did not work. Looked around the webpage.	0	Could not find items related to LGBTQ+ asylum seeker mental health
Canadian Centre for Victims of Torture	https://ccvt.org/	March 8, 2026	LGBT asylum (0 results)	0	N/A
Canadian Centre on Statelessness	http://www.statelessness.ca/	March 8, 2026	LGBT asylum (2 results)	0	None addressed LGBT asylum seeker mental health
Canadian Council of Churches	https://councilofchurches.ca/	March 8, 2026	LGBT asylum (0 results)	0	N/A
Canadian Friends Service Committee - Quaker Committee for Refugees	https://quakerservice.ca/	March 8, 2026	LGBT asylum (1 result)	0	None addressed LGBT asylum seeker mental health
Canadian Labour Congress	https://canadianlabour.ca/	March 8, 2026	LGBT asylum (0 results)	0	N/A
Canadian Refugee Sponsorship Agreement Holders Association (SAH Association)	https://sahassociation.com/	March 8, 2026	No search function provided. Looked around the webpage.	N/A	Could not find items related to LGBTQ+ asylum seeker mental health
Canadian Union of Public Employees (CUPE)	https://cupe.ca/	March 8, 2026	LGBT asylum (15 results)	0	None addressed LGBT asylum seeker mental health

Organization name	URL	Date	Search Terms Used	# items ID'd for full screening	General Comments
Capital Rainbow Refuge	https://capitalrainbow.ca/	March 8, 2026	No search function provided. Looked around the webpage.	1	Had a toolkit for LGBT claims, which can be screened for review
Carty House	https://www.cartyhouse.org/	March 8, 2026	LBGT asylum (0 results)	0	N/A
Catholic Centre for Immigrants, Ottawa	https://cciottawa.ca/	March 8, 2026	LGBT asylum (1 result)	0	None addressed LGBT asylum seeker mental health
Catholic Crosscultural Services	https://ccscan.ca/	March 8, 2026	LGBT asylum (0 results)	0	N/A
Centre de santé communautaire Hamilton/Niagara	https://www.cschn.ca/	March 8, 2026	LGBT asylum (0 results)	0	N/A
Centre for Refugee Studies York University	https://www.yorku.ca/crs/	March 8, 2026	No search function provided. Looked around the webpage.	0	None addressed LGBT asylum seeker mental health
Centre francophone de Toronto	https://www.centrefranco.org/	March 8, 2026	N/A	N/A	Website in French
Chez Marie Refugee Assistance Centre	Website link not found	March 8, 2026	N/A	N/A	N/A
Christie Refugee Welcome Centre	https://christiestreetrc.com/	March 8, 2026	No search function provided. Looked around the webpage.	0	None addressed LGBT asylum seeker mental health

Organization name	URL	Date	Search Terms Used	# items ID'd for full screening	General Comments
Citizens for Public Justice (CPJ)	https://cpj.ca/	March 8, 2026	No search function provided. Looked around the webpage.	0	None addressed LGBT asylum seeker mental health
CLEO (Community Legal Education Ontario / Éducation juridique communautaire Ontario)	https://www.cleo.on.ca/en	March 8, 2026	LGBT asylum (0 results)	0	N/A
Community Legal Services of Ottawa	https://www.clsottawa.ca/	March 8, 2026	LGBT asylum (0 results)	0	N/A
COMPASS Refugee Centre	https://compassrefugee.ca/	March 8, 2026	LGBT asylum (0 results)	0	N/A
Conseil Economique et Social d'Ottawa-Carleton (CESOC)	https://www.cesoc.ca/	March 8, 2026	No search function provided. Looked around the webpage.	N/A	Could not find items related to LGBTQ+ asylum seeker mental health
COSTI Immigrant Services	https://www.costi.org/	March 8, 2026	LGBT asylum (0 results)	0	N/A
CultureLink	http://www.culturelink.ca/	March 8, 2026	No search function provided. Looked around the webpage.	0	None addressed LGBT asylum seeker mental health
Diocese of Hamilton	https://hamiltondiocese.com/	March 8, 2026	LGBT asylum (0 results)	0	N/A

Organization name	URL	Date	Search Terms Used	# items ID'd for full screening	General Comments
Diocese of London - Refugee Ministries	https://www.dol.ca/	March 8, 2026	LGBT asylum (0 results)	0	N/A
Emergency Support Committee for Refugees	https://escr1987.com/home/	March 8, 2026	No search function provided. Looked around the webpage.	0	None addressed LGBT asylum seeker mental health
Exchange Language Services (ELS)	https://exls.ca/	March 8, 2026	No search function provided. Looked around the webpage.	0	None addressed LGBT asylum seeker mental health
FCJ Refugee Centre	https://www.fcjrefugeecentre.org/	March 8, 2026	LGBT asylum (1 result)	0	None addressed LGBT asylum seeker mental health
Focus Humanitarian Assistance Canada	https://focus-canada.org/	March 8, 2026	No search function provided. Looked around the webpage.	0	None addressed LGBT asylum seeker mental health
Fort Erie Multicultural Centre	https://www.forteriemulticulturalcentre.ca/	March 8, 2026	No search function provided. Looked around the webpage.	0	None addressed LGBT asylum seeker mental health
FrancoQueer	https://www.francoqueer.ca/	March 8, 2026	N/A	N/A	Website in French
Huron Refugee Committee, Anglican Diocese of Huron	https://refugee.diohuron.org/	March 8, 2026	No search function provided. Looked around the webpage.	0	None addressed LGBT asylum seeker mental health
Inter Clinic Immigration Working Group (ICIWG)	No website page link found	March 8, 2026	N/A	N/A	N/A

Organization name	URL	Date	Search Terms Used	# items ID'd for full screening	General Comments
International Association for Refugees (IAFR)	https://www.iafr.ca/	March 8, 2026	No search function provided. Looked around the webpage.	0	None addressed LGBT asylum seeker mental health
International Social Service Canada	https://www.issc-ssic.ca/	March 8, 2026	No search function provided. Looked around the webpage.	0	None addressed LGBT asylum seeker mental health
Iraqi Federation of Refugees	https://www.federationcanada.com/en/	March 8, 2026	LGBT asylum (0 results)	0	N/A
Jesuit Refugee and Migrant Service	Duplicate of previous entry	March 8, 2026	N/A	N/A	N/A
Jumpstart Refugee Talent	https://jumpstartrefugee.ca/	March 8, 2026	No search function provided. Looked around the webpage.	0	None addressed LGBT asylum seeker mental health
K-W Multicultural Centre (Kitchener-Waterloo Multicultural Centre)	https://kwmulticultural.ca/refugee-services	March 8, 2026	No search function provided. Looked around the webpage.	0	None addressed LGBT asylum seeker mental health
KAIROS Canada	Broken website link	March 8, 2026	N/A	N/A	N/A
KEYS Job Centre	https://keys.ca/	March 8, 2026	LGBT asylum (0 results)	0	N/A

Organization name	URL	Date	Search Terms Used	# items ID'd for full screening	General Comments
Kingston Community Health Centres- Immigrant Services Kingston & Area	https://kchc.ca/	March 8, 2026	No search function provided. Looked around the webpage.	0	None addressed LGBT asylum seeker mental health
Legal Assistance of Windsor (LAW)	https://www.legalassistanceofwindsor.com/	March 8, 2026	No search function provided. Looked around the webpage.	0	None addressed LGBT asylum seeker mental health
London Cross Cultural Learner Centre (CCLC)	https://www.lcclc.org/	March 8, 2026	No search function provided. Looked around the webpage.	0	None addressed LGBT asylum seeker mental health
MakeWay Charitable Society - Together Project	https://togetherproject.ca/	March 8, 2026	No search function provided. Looked around the webpage.	0	None addressed LGBT asylum seeker mental health
Mary Ward Centre (Ministry of the Loretto Sisters - IBVM Canada)	https://www.marywardcentre.ca/	March 8, 2026	No search function provided. Looked around the webpage.	0	None addressed LGBT asylum seeker mental health
Matthew House - Toronto	https://www.matthewhouse.ca/	March 8, 2026	No search function provided. Looked around the webpage.	0	None addressed LGBT asylum seeker mental health
Matthew House Ottawa: Refugee Services and Furniture Bank	https://www.matthewhouseottawa.org/	March 8, 2026	No search function provided. Looked around the webpage.	0	None addressed LGBT asylum seeker mental health

Organization name	URL	Date	Search Terms Used	# items ID'd for full screening	General Comments
Matthew House Refugee Centre of Windsor	https://matthewhousewindsor.org/	March 8, 2026	No search function provided. Looked around the webpage.	0	None addressed LGBT asylum seeker mental health
Metropolitan Community Church of Toronto - MCC Toronto	https://www.mcctoronto.com/	March 8, 2026	No search function provided. Looked around the webpage.	0	None addressed LGBT asylum seeker mental health. Did have a section of supporting LGBT newcomers.
Micah House Refugee Reception Services	https://www.micahhouse.ca/home	March 8, 2026	No search function provided. Looked around the webpage.	0	None addressed LGBT asylum seeker mental health
National Union of Public and General Employees (NUPGE)	https://nupge.ca/	March 8, 2026	LGBT asylum (0 results)	0	N/A
New Canadians Centre Peterborough	https://nccpeterborough.ca/	March 8, 2026	LGBT asylum (1 result)	0	None addressed LGBT asylum seeker mental health
Newcomer Legal Clinic	https://newcomerlegal.ca/	March 8, 2026	No search function provided. Looked around the webpage.	0	None addressed LGBT asylum seeker mental health
OMRA Shelter Corporation	https://omraottawa.org/	March 8, 2026	No search function provided. Looked around the webpage.	0	None addressed LGBT asylum seeker mental health
Ontario Council of Agencies Serving Immigrants (OCASI)	https://ocasi.org/	March 8, 2026	LGBT asylum (9 results)	4	Looked through webpage for other results as well

Organization name	URL	Date	Search Terms Used	# items ID'd for full screening	General Comments
Ottawa Community Immigrant Services Organization (OCISO)	https://ociso.org/	March 8, 2026	LGBT asylum (2 results)	0	Links in search results were broken
Oxfam Canada	https://www.oxfam.ca/	March 8, 2026	LGBT asylum (1 result)	0	None addressed LGBT asylum seeker mental health
Polycultural Immigrant and Community Service	https://www.polycultural.org/	March 8, 2026	No search function provided. Looked around the webpage.	0	None addressed LGBT asylum seeker mental health
Quinte Immigration Services	https://www.quinteimmigration.ca/	March 8, 2026	LGBT asylum (0 results)	0	
Rainbow Railroad	https://www.rainbowrailroad.org/wp-content/cache/wp-rocket/rainbowrailroad.org/index-https.html	March 8, 2026	Looked into the publications section: 48 results	0	Had publications about LGBTQ+ newcomer and asylum seeker experiences, and mentioned mental health, but was not specific in mental health approaches (only general identifying mental health concerns or used term "mental health supports"
RC Diocese of Thunder Bay (D.O.O.R.S. To New Life - Refugee Centre)	https://www.dotb.ca/	March 8, 2026	LGBT asylum (0 results)	0	N/A

Organization name	URL	Date	Search Terms Used	# items ID'd for full screening	General Comments
Reception House-Waterloo Region	https://receptionhouse.ca/	March 8, 2026	No search function provided. Looked around the webpage.	0	None addressed LGBT asylum seeker mental health
Refugee 613	https://www.refugee613.ca/	March 8, 2026	LGBT asylum (0 results)	0	N/A
Rexdale Community Legal Clinic	https://rexdalecommunitylegalclinic.ca/	March 8, 2026	LGBT asylum (0 results)	0	N/A
Rexdale Women's Centre	https://www.rexdalewomen.org/	March 8, 2026	No search function provided. Looked around the webpage.	0	None addressed LGBT asylum seeker mental health
Romero House	https://romerohouse.org/	March 8, 2026	LGBT asylum (0 results)	0	N/A
Sisters of St. Joseph in Canada - Office for Systemic Justice	https://www.csjcanada.org/	March 8, 2026	No search function provided. Looked around the webpage.	0	None addressed LGBT asylum seeker mental health
Sisters of St. Joseph of Toronto - Ministry for Social Justice, Peace & Care of Earth	https://csj-to.ca/	March 8, 2026	No search function provided. Looked around the webpage.	0	None addressed LGBT asylum seeker mental health
Sojourn House	https://www.sojournhouse.org/	March 8, 2026	LGBT asylum (0 results)	0	N/A

Organization name	URL	Date	Search Terms Used	# items ID'd for full screening	General Comments
Southern Ontario Sanctuary Coalition	Website link unclear and could not find	March 8, 2026	N/A	N/A	N/A
St. Mary's International Charitable Organization	https://stmarysinternational.org/index.html	March 8, 2026	No search function provided. Looked around the webpage.	0	None addressed LGBT asylum seeker mental health
The 519 Church Street Community Centre (The 519)	https://www.the519.org/	March 8, 2026	LGBT asylum (14 results)	3	Programming that supports LGBTQ asylum and refugee claimants
The Canadian Centre to End Human Trafficking (CCEHT)	https://ccteht.ca/	March 8, 2026	No search function provided. Looked around the webpage.	0	None addressed LGBT asylum seeker mental health
The Presbyterian Church in Canada (PCC)	https://presbyterian.ca/pwsd/	March 8, 2026	LGBT asylum (0 results)	0	N/A
The Refugee Hub	Website link no longer active	March 8, 2026	N/A	N/A	N/A
The Synod of the Diocese of Niagara	https://niagaraanglican.ca/	March 8, 2026	LGBT asylum (0 results)	0	N/A
TNO - The Neighbourhood Organization	https://tno-toronto.org/	March 8, 2026	LGBT asylum (0 results)	0	N/A

Organization name	URL	Date	Search Terms Used	# items ID'd for full screening	General Comments
Toronto South Local Immigration Partnership (TSLIP)	https://torontolip.com/	March 8, 2026	LGBT asylum (2 results)	1	Noted a study on LGBTQ asylum seeker mental health
Unifor / Human Rights Department	https://www.unifor.org/	March 8, 2026	LGBT asylum (0 results)	0	N/A
United Church of Canada	https://united-church.ca/	March 8, 2026	LGBT asylum (19 results)	0	None addressed LGBT asylum seeker mental health
Women's Hostels Inc. (Nellie's Women's Shelter)	https://nellies.org/	March 8, 2026	No search function provided. Looked around the webpage.	0	None addressed LGBT asylum seeker mental health
World Education Services (WES)	https://www.wes.org/	March 8, 2026	LGBT asylum (1 result)	0	None addressed LGBT asylum seeker mental health
World Renew	https://worldrenew.ca/	March 8, 2026	LGBT asylum (0 results)	0	N/A
World University Service of Canada (WUSC)	https://wusc.ca/	March 8, 2026	LGBT asylum (0 results)	0	N/A
YMCA of Western Ontario, Windsor Essex Branch	https://www.ymcaswo.ca/	March 8, 2026	LGBT asylum (0 results)	0	N/A

Table S3B

Grey Literature Database Search

Date	Database name	Search strategy(s)	# search results	# brought to screening	General Comments
March 8, 2026	Newcomer Research Library https://newcomerresearch.ca/	LGBT asylum seeker	1	0	Not related to LGBT asylum seeker mental health
		SOGIE asylum seeker	0	N/A	N/A
		SOGIE asylum	0	N/A	N/A
		Queer asylum	0	N/A	N/A

Date	Database name	Search strategy(s)	# search results	# brought to screening	General Comments
March 8, 2026	Government of Canada Library: https://library-archives.canada.ca/eng/collection/Pages/collection.aspx	"SOGIE*" OR "SGM" OR "sexual" OR "gender" OR "LGBT*" OR "2SLGBT*" OR "bisexual" OR "gay?" OR "homosexual" OR "lesbian" OR "men who have sex with men" OR "queer" OR "genderqueer" OR "women who have sex with women" OR "two-spirit" OR "transsexual" OR "transgender" OR "male-to-female" OR "female-to-male" OR "MTF" OR "FTM" OR "asexual" OR "nonbinary" OR "non-cisgender" OR "gender non-conforming" OR "genderless" OR "agender" OR "non-gendered" OR "third gender" OR "same-sex"<br><br>AND<br><br>"asylum" OR "asylee*"<br><br>AND<br><br>"mental" OR "psychological" OR "emotional" OR "community" OR "spiritual" OR "health*" OR "wellness" OR "wellbeing" OR "illness" OR "condition*" OR "support*" OR "diagnosis" OR "resilience" OR "depression" OR "anxiety" OR "stress disorder" OR "suicide" OR “treatment*” or “therap*” or “psychotherap*” or “counsel*” or "psychosocial support" or "psychosocial intervention" or "psychosocial program*"<br><br>AND<br><br>"Canada" OR "British Columbia" OR "Alberta*" OR "Saskatchewan" OR "Manitoba*" OR "Ontario" OR "Quebec" OR "New Brunswick" OR "Nova Scotia" OR "Prince Edward Island" OR "Newfoundland" OR "Labrador" OR "Nunavut" OR "NWT" OR "Northwest Territories" OR "Yukon" OR "Nunavik" OR "Inuvialuit"	37	37	For the search strategy for the Government of Canada Library database, I (A.Y.S.) needed to become more selective of search strings, and I could not use the same search strategy as I had for scholarly databases. This was because I got an "404 - File or directory not found" error and search lines seemed to have a character limit. Therefore, I had used the following aspects of the search process: "All these words" function; Searched in ALL databases; available online; and put a time period of 1991 - 2026.

Table S3C

Google Search Process

Date	Search engine	Search strategy(s)	# items retrieved	# items screened (uploaded for screening)
March 8, 2026	Google Canada	"LGBT asylum claimant mental health supports canada"	The first 50 results	8

Note. Reasons for why literature were not forwarded into the screening process include:

- Duplicates within the Google Search;
- No mention of mental health for SOGIE or LGBTQ+ asylum seekers specifically
- duplicate of item from Canadian Council for Refugees Member Organizations grey literature search;
- literature based outside of Canada (e.g., United States, Australia);
- documents exclusively noting community resources; or
- literature was in video format.

Supplementary Materials File S4) Entities to Chart Descriptions
Table from (So, 2026)

**Criteria**	**Description**
**Covidence ID**	Note the Covidence ID
**Citation**	APA citation of literature
**Year the Study/Literature was Published**	List the year the article/resource was published. "1991", "2001"
**How was literature found**	List<br>• Through systematic search<br>• Through screening references of full-text articles<br>• Through grey literature search
**Study/Literature Aim**	• Do they list the aims/purpose/objectives of the study?
**Number of Participants**	List numerically. "10", "100", "231"
**Participant Age**	List using the following age categories outlined by Statistics Canada (2024):<br>• Children (0-14 years old)<br>• Youth (15-24 years old)<br>• Adults (25-64 years old)<br>• Seniors (65 years and older)<br>• Other (not specified)
**Participant Country of Origin (categorical)**	List the country that participants mentioned are coming from. If not, list (Other: Not specified)
**Participant Country of Origin (numerical)**	List the numbers for each country of origin (if listed)
**Participant Gender Identity (categorical)**	List using the following:<br>• Man<br>• Woman<br>• Non-binary<br>• Trans<br>• Two-spirit<br>• Other (specify)
**Participant Gender Identity (numerical)**	List using numbers for each identity (if listed)
**Participant Sexual Orientation (categorical)**	List using the following<br>• Asexual<br>• Bisexual<br>• Gay<br>• Lesbian

	• Pansexual<br>• Queer<br>• Two-spirit<br>• Other (specify)
**Participant Sexual Orientation (numerical)**	List using numbers for each identity (if listed)
**Region of Canada Indicated**	List the provinces and territories that the study or participants were based in within Canada:<br>• Alberta<br>• British Columbia<br>• Manitoba<br>• New Brunswick<br>• Newfoundland and Labrador<br>• Northwest Territories<br>• Nova Scotia<br>• Nunavut<br>• Ontario<br>• Prince Edward Island<br>• Quebec<br>• Saskatchewan<br>• Yukon
**Study/Literature methods (categorical)**	List using the following:<br>• Quantitative<br>• Qualitative<br>• Mixed methods<br>• Other (specify)
**Study/Literature design**	List the specific quantitative or qualitative method researchers used. Some examples can include:<br>• Community-based participatory research<br>• Narrative Analysis<br>• Grounded Theory<br>• Thematic Analysis
**Data Collection Processes**	List the data collection processes. Some examples include:<br>• Surveys<br>• Interviews<br>• Observations<br>• Online (email)<br>• Online (videoconferencing)
**Mental Health Aspect Addressed (Burdens) (categorical)**	Noting the specific aspect of mental health that was addressed throughout the literature (focused on burdens and challenges). Examples can include:<br>• Anxiety<br>• Belonging<br>• Depression<br>• Discrimination<br>• Gender Identity

	• Homophobia<br>• PTSD<br>• Racism<br>• Suicide<br>• Transphobia<br>• Trauma
**Mental Health Aspect Addressed (Strengths) (categorical)**	Noting the specific aspect of mental health that was addressed throughout the literature (focused on strengths and supports). Examples can include:<br>• Anxiety<br>• Belonging<br>• Depression<br>• Discrimination<br>• Gender Identity<br>• Homophobia<br>• PTSD<br>• Racism<br>• Suicide<br>• Transphobia<br>• Trauma
**Theory/Framework for Approaching Mental Health Support**	Description of what theory or framework the authors used to conceptualize and understand how to approach mental health support for SOGIE asylum seekers and refugee claimants. Some examples include:<br>• Liberation Psychology<br>• Social Determinants of Health<br>• Intersectionality
**Social Determinants of Health Addressed**	If social determinants of health are mentioned, note what social determinants of health were addressed within the study and article. This includes the following from the Public Health Agency of Canada (2024):<br>• Access to health services<br>• Biology and genetic endowment<br>• Childhood experiences<br>• Culture<br>• Education and literacy<br>• Employment and working conditions<br>• Gender<br>• Healthy behaviours<br>• Income and social status<br>• Physical environments<br>• Race / Racism<br>• Social supports and coping skills
**Mental Health Supports and Interventions**	Describe what mental health support and intervention was provided. Some examples of things to comment on include:<br>• Who was it delivered by?

	• What was the method of delivery (online, in-person)?<br>• Where did this support/intervention take place (in community, at a community organization, hospital, NGO, schools, etc…)<br>• Who delivered the mental health support/intervention (settlement worker, peers, etc…)
**Evaluation and Outcomes of Mental Health Support/Intervention**	Describe what was evaluated and outcomes of the mental health support/intervention.
**Study/Literature Results/Findings about Mental Health Supports and Interventions**	List the study results and findings related to mental health, mental health supports, and mental health interventions.
**Study/Literature Limitations/Considerations**	List the study limitations and considerations mentioned in the literature.
**Study/Literature Suggestions for Practical Implications/Recommendations about Mental Health Supports and Interventions**	List the suggestions for practical implications and recommendations mentioned in the literature related to mental health.
**Study/Literature Suggestions for Future Research**	List the future directions that the literature identified.
**General Comments**	Any general comments from the extractor.
